# Factor interaction analysis for chromosome 8 and DNA methylation alterations highlights innate immune response suppression and cytoskeletal changes in prostate cancer

**DOI:** 10.1186/1476-4598-6-14

**Published:** 2007-02-05

**Authors:** Wolfgang A Schulz, Adrian Alexa, Volker Jung, Christiane Hader, Michèle J Hoffmann, Masanori Yamanaka, Sandy Fritzsche, Agnes Wlazlinski, Mirko Müller, Thomas Lengauer, Rainer Engers, Andrea R Florl, Bernd Wullich, Jörg Rahnenführer

**Affiliations:** 1 Department of Urology, Heinrich Heine University, Düsseldorf, Germany; 2 Max-Planck Institute for Informatics, Saarbrücken, Germany; 3 Department of Urology, Medical University of the Saarland, Homburg, Germany; 4 Institute of Pathology, Heinrich Heine University, Duesseldorf, Germany

## Abstract

**Background:**

Alterations of chromosome 8 and hypomethylation of LINE-1 retrotransposons are common alterations in advanced prostate carcinoma. In a former study including many metastatic cases, they strongly correlated with each other. To elucidate a possible interaction between the two alterations, we investigated their relationship in less advanced prostate cancers.

**Results:**

In 50 primary tumor tissues, no correlation was observed between chromosome 8 alterations determined by comparative genomic hybridization and LINE-1 hypomethylation measured by Southern blot hybridization. The discrepancy towards the former study, which had been dominated by advanced stage cases, suggests that both alterations converge and interact during prostate cancer progression. Therefore, interaction analysis was performed on microarray-based expression profiles of cancers harboring both alterations, only one, or none. Application of a novel bioinformatic method identified Gene Ontology (GO) groups related to innate immunity, cytoskeletal organization and cell adhesion as common targets of both alterations. Many genes targeted by their interaction were involved in type I and II interferon signaling and several were functionally related to hereditary prostate cancer genes. In addition, the interaction appeared to influence a switch in the expression pattern of *EPB41L *genes encoding 4.1 cytoskeleton proteins. Real-time RT-PCR revealed *GADD45A*, *MX1*, *EPB41L3*/*DAL1*, and *FBLN1 *as generally downregulated in prostate cancer, whereas *HOXB13 *and *EPB41L4B *were upregulated. *TLR3 *was downregulated in a subset of the cases and associated with recurrence. Downregulation of *EPB41L3*, but not of *GADD45A*, was associated with promoter hypermethylation, which was detected in 79% of carcinoma samples.

**Conclusion:**

Alterations of chromosome 8 and DNA hypomethylation in prostate cancer probably do not cause each other, but converge during progression. The present analysis implicates their interaction in innate immune response suppression and cytoskeletal changes during prostate cancer progression. The study thus highlights novel mechanisms in prostate cancer progression and identifies novel candidate genes for diagnostic and therapeutic purposes. In particular, *TLR3 *expression might be useful for prostate cancer prognosis and *EPB41L3 *hypermethylation for its detection.

## Background

Up to 40% of all elderly men may harbor prostate carcinomas, less than 20% develop symptomatic disease, which in about 3% becomes the cause of death. Great advances have been made in prostate cancer detection due to refined PSA-based assays, imaging and histopathology. In their wake, two questions have gained importance, i.e. which tumors represent clinically significant disease and how tumors having progressed locally or metastasized can be identified and appropriately treated. Molecular research is thus challenged to provide a reliable classification of prostate cancers and to identify targets for novel therapies in those tumors no longer containable by surgery, irradiation, and anti-androgenic therapy.

Among such aggressive prostate cancers, molecular alterations are not uniform. Instead, these tumors contain different combinations of genetic and epigenetic aberrations that each appear to influence the biological processes crucial for the cancer phenotype [1-3]. It is therefore necessary to disentangle the influence of individual alterations by factor analysis approaches. Through defining the biological consequences of particular recurrent molecular alterations – considered as factors – it may become possible to identify subgroups of prostate carcinoma whose behavior is determined by them. These factors could then be used to predict the prognosis in each particular subgroup. In addition, elucidation of the mechanisms by which a factor exerts its effects on prostate cancer progression would provide specific targets for therapy within the respective subgroup. This approach does not imply that factors act independently of each other. Indeed, we suggest here that two molecular alterations previously identified as being associated with more aggressive prostate cancers appear to interact in a synergistic fashion during tumor progression.

The first factor, alteration of chromosome 8, is found in up to 50% of prostate cancers [1-3]. A large number of publications have concordantly reported that chromosome 8 alterations are significantly associated with various established histopathological indicators of poor prognosis or directly with clinical outcome. Curiously, 8p losses or 8q gains both seem to have similar clinical effects (reviewed in [4]). One reason for this unusual relationship is that 8q gain is often mechanistically linked to 8p loss through isochromosome 8q formation [5]. Moreover, it has been difficult to trace the effect of chromosome 8 alterations to any single gene [2]. Interestingly, chromosome 8 has also emerged as the site of a hereditary prostate cancer gene in genome-wide searches. A good candidate is *MSR1 *at 8p23.1. Inherited mutations at this locus are presumed to modify immune responses during carcinogenesis [1]. Thus, the importance of chromosome 8 alterations as a factor in prostate cancer progression is undisputed, but the question of which biological processes they influence is open.

The second factor is hypomethylation of LINE-1 retrotransposons, which constitute approximately 18% of the human genome. LINE-1 hypomethylation is part of a broader process, 'genome-wide' hypomethylation that affects not only repeat sequences, but also single-copy genes inactivated by DNA methylation in normal adult tissues [6]. While hypomethylation occurs at early stages in some cancer types, it is associated with tumor progression in others. Conceivably, this association is brought about by increased chromosomal instability as a consequence of repetitive sequence hypomethylation and by reactivation of specific genes favoring tumor cell survival and adaptation during invasion and metastasis [7]. However, the details of the relationship between genome-wide hypomethylation and cancer progression are poorly understood. In prostate cancer, LINE-1 hypomethylation is detected in only a subset of localized cancers, but is highly prevalent in high-stage and metastatic cases [8,9]. In contrast, hypermethylation of selected single copy genes occurs consistently at early stages of carcinogenesis [9,10].

The two factors, chromosome 8 alterations and LINE-1 hypomethylation, may not be independent of each other. In a previous study of 55 prostate carcinomas including many advanced stage and recurrent cases, we found LINE-1 hypomethylation to be highly significantly associated with chromosomal instability [8], in keeping with expectations from other cancer types and animal models [6,7]. Unexpectedly, we found a particularly close relationship between hypomethylation and chromosome 8 alterations. This raised the question whether this association was (i) mechanistic, i.e. a consequence of chromosome 8 destabilization by hypomethylation or, conversely, deregulation of DNA methylation as a consequence of chromosome 8 changes, (ii) the consequence of a common factor causing both alterations, or (iii) an indication of a functional interaction between both factors that promotes prostate cancer progression. Here, we will present evidence that explanation (iii) is most likely. Our analysis revealed that the interaction of the two factors appears to relate to several biological processes already implicated in prostate cancer progression, prominently suppression of immune responses and changes in the cortical actin cytoskeleton and the extracellular matrix.

## Results

### Comparative genomic hybridization and DNA methylation analysis

Our previous study had revealed a highly significant association between LINE-1 hypomethylation and chromosome 8 alterations, particularly in tumors with distant metastases or recurring after anti-androgenic therapy [8]. To further elucidate this association, we investigated whether it also existed in less advanced cancers. From a previous study of DNA methylation alterations in prostate carcinoma [9], a subset of 50 primary tumors without distant metastases were selected by the criteria of availability of high-quality DNA and RNA and complete follow-up data (Table 1). Almost all tumor tissues presented hypermethylation in several genes typically hypermethylated in prostate cancer (Table 1).

**Table 1:** Clinical, methylation and CGH data for prostate cancer tissues

Tumor No.	Stage	Gleason Score	Molecular Group	LINE-1 %hypo	No. hypem. genes	Chromosomal losses	Chromosomal gains
36	pT3bN0	7		1	4	-	3q21q26.2
38	pT2N0	7		4	4	-	-
**50**	pT3bN0	7	hypo	**10**	4	1q31	6p,7p,16p,17p, 18p,19, 20q13,21,22,Y
**65**	pT3bN0	7	both	**9**	4	Y	1p33p34.3, 2p21p23, 2q14.1q14.3, 2q33q36, 3p14.1p21.3, 3q25.1q26.3, 4q13.3q28, 5q22, 5q31.2q32, 6q16.1q22.1, **8q22.1q23**, 10p11.2p13, 10q11.2q22.3, 12q13.2q24.31, 13q21.1q31, 14q21q32.2, 18q11.2q22, Xp21.3q27
**83**	pT3bN0	7	hypo	**6**	4	1q31, 2q33q37, 4q31, 13q21q31, 15q24,	4q12, 5q13, 17p, 18p, 19, Y
**89**	pT2bN0	3	hypo	**5**	2	9q33q34, X	2q11q24, 4q12q22, 6q22, 12p,18p, Y
93	pT3bN0	7		4	4	-	Yq
**95**	pT3bN1	10	hypo	**7**	4	Xq	18p, Y
99	pT2bN0	5		0	1	-	2q14q24,10q11q21
**101**	pT3aN0	8	none	0	3	1p36, 14q31q32, 17p, 22	-
105	pT3aN0	5		1	1	1p36,2,3,4	-
**107**	pT3aN0	7	none	3	4	18p, Y	4q24, 6q22, Xp11.2p11.4
117	pT3bN0	5		3	4	-	1q21q25,2q11q14,19q13
**119**	pT3bN1	9	none	1	3	7p15pter, 15q23qter, Y	10q21q22
**121**	pT2bN0	6	chrom8	1	4	1q32q41, 3p21, 4p15p16, **8p22p23**, 9p23p24,10q25q26, 11q23q25, 12q24, 13q32q34, 14q24q32, 15q24q26, 17q24q25, Xp21p22	Yq
123	pT2aN0	5		1	1	9q21.2q21.3, 10q26, 16p, Y	2p14p16, 2q24.3q32.1, 6q14q16.1
125	pT2bN0	6		1	0	14q31	-
**127**	pT2bN0	7	none	2	2	7q36, Yq	2q22q32.3, 4q11q31.3, 13q14.1q21.1, 14q21q23, 15q21.1q23
**133**	pT2bN1	7	chrom8	0	4	22, Y	1q, 2q22q34, 3q11.1q26.1, 4q22q27, 5q21q31, 6q21q24, **8q11q23**, 10q11q21.3, 12q21q22, 14q12q22, X
**137**	pT2bN0	8	chrom8	1	1	16, 17, 18p, 18q11.1q21.1, 19, 21q22, Y	3q23q26.3, 4q13.2q21.3, 5q, 7q13q31, 8p21pter, **8q24**, 9p, 14q13qter
**139**	pT3bN1	9	chrom8	1	4	19q, Y	1q25q31, 2q11q33, 3, 4q, 5q13q23, 6q14q25, 7q21qter, **8q13q23**, 11q14qter, 13q21q32, 14q11.1q21, 18q12, Xq21.3qter
**141**	pT2bN0	4	chrom8	0	2	16, 17, 18p, 19, 20, 22, Y	1q31q32.1, 2, 3q13.3qter, 4q11q31.1, 5q14qter, 6q, 7p12pter, 7q22q31.2, **8q12q21.2**, 10q21.1q23.1, 11q14.3qter, 12q14qter, 13q14.1qter
**145**	pT4N1	7	hypo	**11**	3	Y	3q25q27,4q24q26,7q11q22,13q31q34,17p,18p
149	pT2bN0	6		2	2	Y	2p, 4p12, 4q24q26, 5q11q13, 7p12, **8q23q24**, 9,11q22, 12q12q15, 14q24, 17, 19, 20q13,
151	pT3aN0	7		0	1	3p13p14	6p14, Xp21
153	pT3aN0	5		2	1		Xq11.2q13
155	pT3aN0	5		**5**	4	19p	3p25p26, Y
157	pT2aN0	8		2	0	-	1q21q31, 2q12q24, 3q, 4q12q21, 5q13q14, 6q14q16, 7q11, **8q12q22**, 12q12q13, 18p, Y
161	pT2bN0	5		3	4	15q22q24, Xq22q25	-
**163**	pT3bN1	5	chrom8	2	2	16, 17p, 18p, 20, Y	1q25qter, 2p11p16, 2q32q34, 3q12, 3q21q27, 4q, 6q15q24, **8q12q21**, **8q23q24**, 12q13q21, 13q14q32, 14q12q21, 18q, Xq11.1q26
**169**	pT3aN0	7	chrom8	3	2	16, 17p, 18p, 19, Y	2, 3, 4q, 5q, 7p15pter, **8q22q24**, 13q31qter, 14q24qter, 17q23, 18q21qter, Xq21.3q26
**171**	pT2bN0	5	hypo	**8**	4	10q26, 18p. -19, 22, Y	1p31, 2p12p13, 2q11q34, 4q22q26, 5q22, 6q11q23, 14q22q24, X
175	pT2bN0	8		**11**	4	13q14	-
183	pT3aN0	6		**19**	4	1p36,9q34	-
**187**	pT2bN0	8	chrom8	1	3	16, 17p, 18p, 19, 21, 22, Y	1, 2, 3q13qter, 4q, 5q14q33.1, 6q16q23, 7p, 7q22q33, **8q**, 11q14.3qter, 12q14qter, 13q21qter, 14q24q31, 15q, Xq22qter
**189**	pT2bN0	7	none	0	4	3p25p26, 10q26, Xq22qter, Yq	-
191	pT2bN0	7		0	3	4q31, 5q31q35, 9q33, 12p12	-
205	pT3aN0	7		**5**	1	18p, 19, 22, Y	2q31q33, 6q22.3q23, 10q21q22, Xp11.1p11.4, Xq
**209**	pT3aN0	7	both	**8**	4	1p36, **8p**, 16, 17, 18p, 19, 20, 22, Y	1q31q32.1, 2q11.1q34, 3, 4q21.1qter, 5q14q31, 6q11q23, 7q21q34, **8q**, 9, 11q14q22, 12q15q24, 13q14qter, 18q
213	pT2aN0	7		**8**	4	8q24.2qter, 14q32, 16, 17, 18p, 22, Y	-
215	pT3aN0	7		**9**	4	19q13, 22q13,Y	3q24q26, 5q12, 8p12, 17, 18, Xp11p21, X21q24
217	pT2bN0	8		**5**	4	18p13, Y	2q33q35, 4q13q28,5q21q31,**8q21,8q24**,12q13, 12q21q23, Xq13q25
**219**	pT4N1	7	both	**21**	4	5q34qter, **8p22pter**, 19, Yq	1p22p31.3, 4q21q22, 4q31.1, 5q13, 6q24q26, 7q, **8q21q24**, 10q21, 11q14q22, 20q13, Xq13q22
225	pT3bN0	6		4	4	4q28, 8q24qter, Yq	-
227	pT2aN1	7		3	4	22q13	3q11q21
**232**	pT2bN1	7	both	**13**	4	**8p22pter**	-
245	pT3aN0	7		4	3	17p13, 18p	1p31
**247**	pT3bN1	7	chrom8	3	3	**8p21pter**, 9q21, 22, Y	Xq12
253	pT3aN1	7		1	4	1p36, 2q33q37, 6p21.3p25, **8p21**, 14q31q32, Y	-
256	pT3bN0	7		1	4	2p2p21, 6q, 15q26,16p, 18,X	-

The numbers of the tumors used in the microarray analysis are in bold type, as are hypomethylation values considered significantly elevated (< 4%) and typical chromosome 8 aberrations. All cancers were M0 at the time of prostatectomy. No. hypermethylated genes indicates hypermethylation of *APC*, *GSTP1*, *RARB2*, and *RASSF1A*. LINE-1 hypomethylation was determined by Southern blot analysis. Chromosomal dosage changes were determined by comparative genomic hybridization.

For all cases, comparative genomic hybridization (CGH) analysis was performed using the same DNA aliquots employed in methylation analysis (Table 1). Overall, 17 tumors revealed typical changes in chromosome 8 dosage. Loss of 8p was detected in 6 cases and gain of 8q in 13 cases. Three tumors exhibiting nontypical 8p gain or 8q loss were not categorized as 'chromosome 8 altered'.

Significant LINE-1 hypomethylation (>4%) was found in 17 tumor specimens (Table 1). Only five of these harbored 8p losses or 8q gains, 12 tumors each contained either hypomethylation or chromosome 8 alterations, and in 21 cases neither alteration was detected. Thus, in the present series no association was found between LINE-1 hypomethylation and chromosome 8 alterations. The previously measured association therefore likely reflects a convergence in late stage prostate cancers, i.e. both molecular alterations may be selected for during progression of prostate cancers towards an aggressive phenotype. This co-selection is most straightforwardly explained by a synergistic influence of the two factors on biological processes important for tumor progression.

### Microarray expression analysis

To identify which biological processes might be targeted by the supposed synergism, expression profiling was performed. From those cases for which ample high quality RNA was available, 24 tumors were selected exhibiting only chromosome 8 alterations (8p loss, 8q gain, or both), only LINE-1 hypomethylation, chromosome 8 alterations as well as LINE-1 hypomethylation ('*both*' group), or neither alteration (Table 1). The specimens lacking both changes were selected to harbor a maximum of other chromosomal alterations. Total RNA from each case was hybridized to Affymetrix U133A oligonucleotide arrays, without pre-amplification.

This experimental setup allows three comparisons, i.e. (i) cancers with or without chromosome 8 alterations, (ii) cancers with or without LINE-1 hypomethylation, and (iii) an interaction analysis of both factors. In the interaction analysis, we considered those genes as significant, whose changes in expression were significantly greater in '*both' *cancers than the sum of the changes in cancers with one alteration, each compared to the cancers without either alteration (see Methods). Moreover, since we were interested in the identification of biological processes targeted by the presumed interaction, all comparisons were performed for Gene Ontology (GO) 'biological process' groups (see Methods). In this analysis, a Gene Ontology group is highlighted if it is enriched, i.e. if it contains statistically significantly more differentially expressed genes than expected by chance.

**Figure 1:**
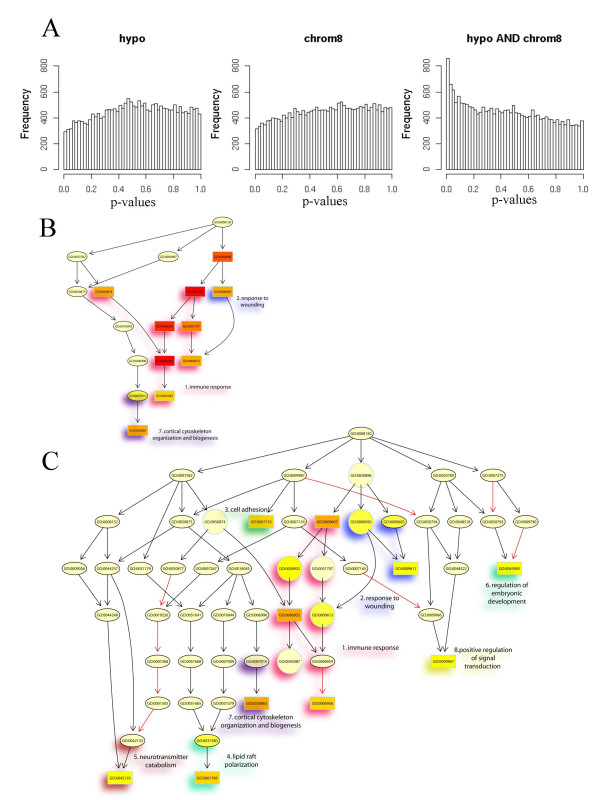
**Bioinformatic analysis of microarray expression data**. **A: **The distributions of the raw p-values of all genes for the main effects *hypomethylation of LINE-1 retrotransposons *(hypo), *alteration of chromosome 8 *(chrom8) and for the *interaction effect *(hypo AND chrom8). In each graph the numbers of genes with p-values in the indicated ranges (in increments of 0.02) are shown. The uniform distributions of the p-values for the two main single effects indicate that not more genes are declared significant than expected at random, whereas for the interaction effect a skewed distribution is observed, i.e. significantly small p-values are assigned to a large number of genes. **B: **The subgraph induced by the top 15 GO terms identified by the *classic *algorithm for scoring GO terms for enrichment. In this graph, nodes represent GO terms and edges represent parent-child relationships, i.e. an arrow from node A to node B indicates that the genes in B are a subset of the genes in A. Black arrows indicate is-a relationships and red arrows part-of relationships, as defined in the Gene Ontology nomenclature. In general, GO terms are represented by ellipses with the corresponding GO IDs plotted inside. GO IDs surrounded by boxes instead of ellipses indicate the 10 most significant GO terms as identified by enrichment analysis. Color represents the relative significance of enrichment, ranging from dark red (most significant) to light yellow (least significant). Interesting areas in the GO graph defined by significant related GO terms are highlighted by different underlying colors (e.g. red for immune response). **C: **The subgraph induced by the top 15 GO terms identified by the *weight *algorithm for scoring GO terms for enrichment. For a detailed description see Fig. 1B. Circles instead of ellipses indicate GO terms that are found significant by the *classic *algorithm but not by the *weight *algorithm. see Additional file 3 for a listing of all GO groups in Fig. 1B and 1C

Intriguingly, few differences were discovered in the comparisons based upon differential methylation or upon the presence of chromosome 8 alterations (Figure 1A). In contrast, the interaction analysis revealed a number of highly significant differences (Figure 1A). Initially, the *classic *algorithm (see Methods) for testing enrichment was used. The majority of the significant terms obtained with this algorithm were related to 'immune response' and 'response towards wounding' (areas 1. and 2. in Figure 1B). A prominent unrelated GO group was #30865 (area 7. in Figure 1B). Individual genes significant within GO groups are listed in Additional file 1 (further information on Figures 1B and 1C can be found in Additional file 3).

### Expression of immune response-related genes

**Figure 2:**
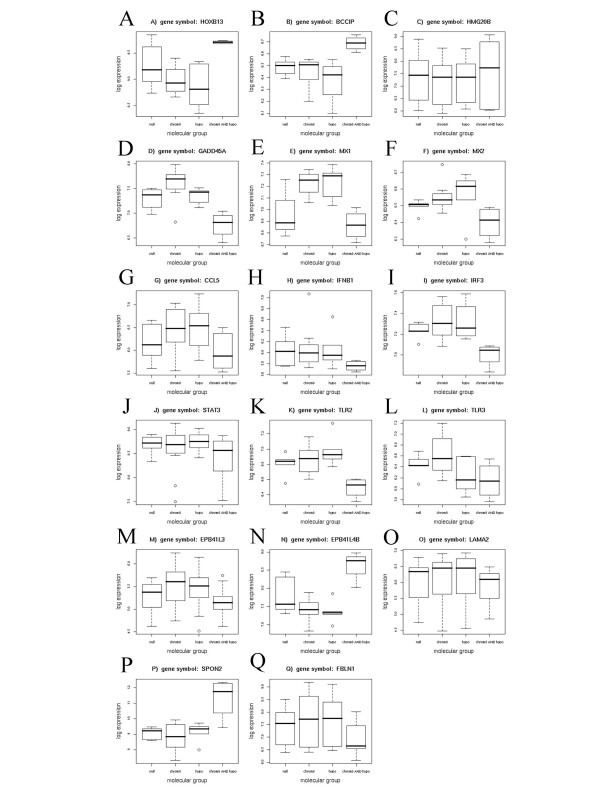
**Box plot representation of microarray analysis results for selected genes**. **A**: *HOXB13*; **B**: *BCCIPA*; **C**: *HMG20B*; **D**: *GADD45A*; **E**: *MX1*; **F**: *MX2*; **G**: *CCL5*; **H**: *IFNB1*; **I**: *IRF3*; **J**: *STAT3*; **K**: *TLR2*; **L**: *TLR3*; **M**: *EPB41L3*; **N**: *EPB41L4B*; **O**: *LAMA2*; **P**: *SPON2*; **Q**: *FBLN1*. In each graph, the expression values (log-scale) for the respective genes are depicted for the cancers with neither hypomethylation of LINE-1 retrotransposons nor alteration of chromosome 8 (null), hypomethylation only (hypo), alteration of chromosome 8 only (chrom8) and both alterations (chrom8 AND hypo). The p-values refer to the result of the interaction analysis (see Methods for details).

**Figure 3:**
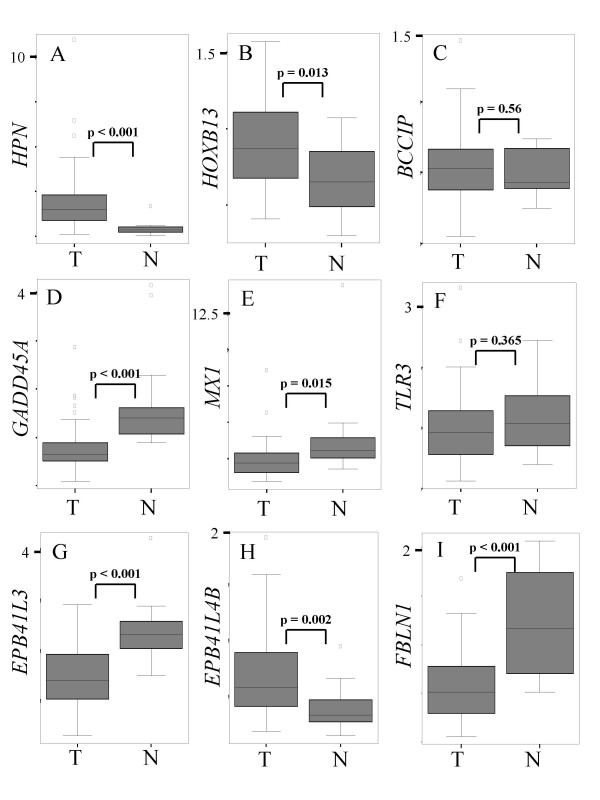
**Box plot comparison of expression of selected genes in prostate cancer vs. benign tissues by real-time quantiative RT-PCR**. **A**: *HPN*; **B**: *HOXB13*; **C**: *BCCIP*; **D**: *GADD45A*; **E**: *MX1*; **F**;*TLR3*; **G**: *EPB41L3*; **H**: *EPB41L4B*; **I**: *FBLN1*. Expression values were determined for each sample in duplicate with < 10% variation. They are indicated relative to the reference gene *TBP *determined in the same fashion. T: cancer samples (n = 47), N: benign tissue samples (n = 13); p-values according to Mann-Whitney tests.

Several genes individually significant in the interaction analysis were selected for closer analysis. The microarray results for these and some related other genes are shown in Figure 2. Figure 3 presents a comparison of their expression analyzed by quantitative RT-PCR in 47 cancer samples (including almost all used in the microarray analysis) and 13 morphologically normal prostate tissues obtained from distant sites of cancer-carrying prostates. This latter analysis allows to determine whether these genes were differentially expressed not only among prostate cancer groups, but also between prostate cancers and noncancerous tissue in general. As a quality control, *HPN *(Hepsin) expression was first determined in all these samples and was strongly increased in cancerous over benign tissues (Figure 3A), as expected [14-17]. As another quality control, DNA from the benign tissues used exhibited neither *GSTP1 *nor *RARB2 *hypermethylation, excluding contamination with tumor [9,10].

Like Hepsin, *HOXB13 *expression was overall significantly elevated in cancer tissue (Figure 3B), although the increase was highly variable. This variation was obviously related to the presence of chromosome 8 alterations and LINE-1 hypomethylation (Figure 2A). No other classical homeobox gene was significant, including the closest *HOXB13 *paralogs, *HOXC13 *and *HOXD13 *(data not shown). The upregulation of *HOXB13*, but also its variability are in keeping with previous studies [17,19].

In contrast, *BCCIP *was identified as significant (p = 0.0036) in the interaction analysis, with maximum expression in the '*both*' group (Figure 2B), but its expression did not differ significantly between cancer and noncancerous tissues overall (Figure 3C). BCCIP interacts with BRCA2. Comparisons of *BRCA1*, *BRCA2*, and *BARD1 *did not yield differences (data not shown), but another gene interacting with *BRCA2*, *HMG20B*, was significant in the interaction analysis (Figure 2C). Altered expression of *HMG20B *has previously been reported in prostate cancer [18].

*GADD45A*, which is also induced by BRCA-dependent pathways, was significantly decreased in prostate cancers compared to noncancerous tissues (Figure 3D), with the lowest levels in '*both*' cancers (Figure 2D). Expression of *GADD45B *or *GADD45G *was not significantly different between the tumor groups.

Several genes related to interferon signaling were downregulated in the '*both*' cancers, most significantly, the prototypic type I interferon response gene *MX1 (*also named *MXA*) (Figure 2E). *MX2 *(*MXB*) behaved similarly (Figure 2F). *MX1 *was also downregulated in cancer tissues overall (Figure 3E). A significant decrease in the '*both*' group was also observed for *CCL5 *encoding a chemokine enhancing interferon responses (Figure 2G). Expression of *IFNB1 *(encoding IFNβ), but not of *IFNG *or *IFNA *genes, was significantly lowered according to the interaction analysis (Figure 2H), and so was accordingly *IRF3 *(Figure 2I) encoding a major transcriptional activator of *IFNB1*. Another important transcription factor for type I interferons, *STAT3*, showed minor decreases (Figure 2J). Interestingly, selected TLRs were downregulated in '*both' *cancers, especially *TLR2 *(Figure 2K) and *TLR3 *(Figure 2L). *TLR3 *expression was decreased in many, but not all prostate cancers compared to normal tissues (Figure 3F).

The expression differences in immune-response genes might potentially be due to altered proportions of innate immune cells in the different tumor groups. We therefore analyzed, whether several typical marker genes of NK cells, mast cells, macrophages, dendritic cells, and granulocytes were differentially expressed in the '*both*' cancers [see Additional File 2]. Only a single marker, *ITGAM (more commonly known as MAC-1*) showed a significant, but small difference between the groups. From this analysis, the differences in the proportions of innate immune cells between the tumor groups appear to be small and cannot account for the overall observed expression differences.

### Expression of cytoskeleton and extracellular matrix genes

Since the *classic *algorithm for detecting enriched GO terms treats each GO term independently, related biological terms often appear simultaneously among the top scoring significant GO terms. In the more sophisticated *weight *algorithm, genes annotated to a GO term receive weights based on the significance of neighboring GO terms (see Methods). This approach identifies local dependencies between related GO terms and highlights those terms that receive a more significant enrichment score than all their neighbors in the GO graph. Upon application of the *weight *algorithm the most significant GO terms are spread over additional areas in the GO graph (compare Figure 1B and 1C), identifying a larger variety of biological processes (Table 2), in addition to those related to immune response. Most significantly, the *weight *algorithm highlights another set of GO groups concerning the interacting processes of cortical actin cytoskeletal organization and cell adhesion (areas 3. and 7. in Figure 1C). In fact, the significance of GO groups 'regulation of embryonic development' and 'positive regulation of signal transduction' (areas 6. and 8. in Figure 1C) also derives predominantly from genes related to cell adhesion [see Additional file 1].

**Table 2:** Top 20 significant GO groups in interaction analysis, sorted according to the *weight *algorithm

Rank (weight)	GO ID	GO Term definition	Annotated number of genes	Significant number of genes	Expected number of genes	Rank (classic)	p-value (classic)	p-value (weight)
1	GO:0006955	immune response	1241	57	26.05	2	1.50E-08	3.30E-05
2	GO:0009607	response to biotic stimulus	1409	63	29.58	1	6.40E-09	5.30E-05
3	GO:0030865	cortical cytoskeleton organization and biogenesis	22	5	0.46	8	7.80E-05	7.80E-05
4	GO:0001766	lipid raft polarization	2	2	0.04	11	0.00044	0.00044
5	GO:0006956	complement activation	49	6	1.03	15	0.00054	0.00054
6	GO:0007155	cell adhesion	972	36	20.40	16	0.00067	0.00076
7	GO:0009967	positive regulation of signal transduction	176	11	3.69	22	0.00125	0.00125
8	GO:0009611	response to wounding	589	25	12.36	17	0.00070	0.00137
9	GO:0045995	regulation of embryonic development	12	3	0.25	24	0.00175	0.00175
10	GO:0042135	neurotransmitter catabolism	15	3	0.31	28	0.00346	0.00346
11	GO:0043122	regulation of I-kappaB kinase/NF-kappaB cascade	149	9	3.13	29	0.00425	0.00425
12	GO:0006952	defense response	1354	60	28.42	3	2.20E-08	0.00445
13	GO:0009190	cyclic nucleotide biosynthesis	33	4	0.69	33	0.00483	0.00483
14	GO:0050678	regulation of epithelial cell proliferation	17	3	0.36	34	0.00501	0.00501
15	GO:0008354	germ cell migration	7	2	0.15	39	0.00861	0.00861
16	GO:0009613	response to pest, pathogen or parasite	776	34	16.29	7	4.20E-05	0.01258
17	GO:0016064	humoral defense mechanism (sensu Vertebrata)	165	10	3.46	25	0.00258	0.01310
18	GO:0006024	glycosaminoglycan biosynthesis	24	3	0.50	46	0.01339	0.01339
19	GO:0050672	negative regulation of lymphocyte proliferation	9	2	0.19	47	0.01435	0.01435
20	GO:0008037	cell recognition	28	3	0.59	58	0.02038	0.02038

The conspicuous GO group #30865 comprises mostly genes encoding band 4.1 proteins, officially termed *EPB41L*. Most family members were down-regulated in the '*both*' cancers, especially *EPB41L3 *(Figure 2M) encoding 4.1B. Its expression was highly significantly downregulated in all prostate cancers compared to normal tissues (Figure 3G). In contrast, a more distant paralog, *EPB41L4B *encoding EHM2, was strongly upregulated in the '*both' *group (Figure 2N), and in cancers overall (Figure 3H). Expression of *EPB41L3 *and *EPB41L4B *correlated inversely with each other (Pearson -0.574, p < 0.0001).

A related group of genes identified by interaction analysis encodes components of basement membranes and extracellular matrix. Genes encoding laminin subunits (Figure 2O) and spondins (Figure 2P) have previously been identified as differentially expressed in prostate cancer [14-17,19]. In addition, we observed *FBLN1 *encoding the basement membrane component Fibulin-1 to be strongly downregulated in '*both' *cancers (Figure 2Q), and in cancers overall (Figure 3I).

In addition to GO groups related to immune response and cell adhesion or whose significance derives from expression differences in genes belong to these larger categories, interaction analysis by the *weight *algorithm revealed several others (Figure 1C, Table 2), which were not investigated further here. Interestingly, many genes significant in these groups have previously been implicated in prostate cancer. For instance, GO group #43122 contains genes related to NFκB regulation. Constitutive activation of NFκB is widespread in prostate cancer and likely associated with progression (reviewed in [20]). Increased activity of Gα proteins and PKA (GO #9187) is thought to enhance androgen receptor activity [21] and influence hedgehog signaling [22]. Expression of monoamine oxidase responsible for the significance of GO group #42135 correlates closely with Gleason score and prostate cancer prognosis [23]. Phosphatic acid phosphatase 2a responsible for the significance of the GO group #8354 was previously identified in a screen for genes differentially expressed in prostate cancer [24].

For all genes in Fig. 3 the relationship of expression to tumor stage, lymph node involvement, Gleason score, and biochemical recurrence was investigated. After adjustment for multiple testing, significant associations were obtained for two relations. *HPN *expression was associated with Gleason score, and *TLR3 *expression was associated with recurrence. As for several other genes, altered expression of *TLR3 *tended furthermore to be associated with increased tumor stage.

### DNA methylation analysis of novel prostate cancer genes

Several genes conspicuous in the present study are subject to DNA hypermethylation in other cancers, prominently *EPB41L3 *[25,26] and *GADD45A *[27].

**Figure 4:**
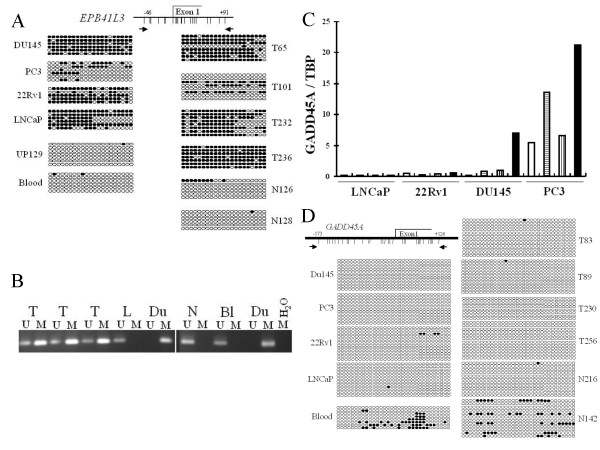
**DNA methylation analysis of *EPB41L3 *and *GADD45A***. **A**: Bisulfite sequencing of the *EPB41L3 *promoter in selected cell lines and prostate tissue samples. Du145, PC3, 22Rv1, and LNCaP: prostate cancer cell lines. T: tumor tissues; N: benign tissues, UP: normal urothelial cells. **B**: Examples of Methylation-specific PCR for *EPB41L3*. M: primers specific for methylated promoter sequence, U: primers specific for unmethylated promoter sequence. T: prostate carcinoma tissue, N: benign prostate tissue, Bl: blood leukocyte DNA as unmethylated control, Du: Du145 DNA as methylated control. **C**: Effect of 5-aza-dC and SAHA on *GADD45A *expression. For each cell line from left to right: untreated (white bars), 5-aza-dC (horizontal stripes), SAHA (vertical stripes), 5-aza-dC + SAHA (black bars). **D**: Bisulfite sequencing of *GADD45A *in selected cell lines and prostate tissue samples. Du145, PC3, 22Rv1, and LNCaP: prostate cancer cell lines. T: tumor tissues; N: benign tissues

Downregulation of *EPB41L3 *in other carcinomas is caused by allelic loss at 18p11.3 or promoter hypermethylation [25,26]. In the present study, eleven cancers showed loss, but only 4 gains at chromosome 18p (Table 1). Bisulfite sequencing revealed *EPB41L3 *promoter hypermethylation in prostate cancer tissues and cell lines, low methylation in benign prostate tissues and none in leukocytes (Figure 4A). Methylation-specific PCR (Figure 4B) detected *EPB41L3 *hypermethylation in 79% of the cancer tissues. In prostate carcinoma cell lines, with the exception of PC3, *EPB41L3 *expression was undetectable even by highly sensitive real-time RT-PCR. Accordingly, the *EPB41L3 *promoter was strongly methylated in all cell lines, but less so in PC3 (Figure 4A). Combined treatment with a DNA methyltransferase inhibitor, 5-aza-dC, and a histone deacetylase inhibitor, SAHA, induced *EPB41L3 *expression to detectable levels in Du145 and 22Rv1, while expression in PC3 and LNCaP remained unchanged (data not shown). In the same experiment, 5-aza-dC strongly induced *CTCFL *and SAHA induced *CDKN1A*/p21^CIP1 ^expression, as described ([11]).

Treatment with a combination of 5-aza-dC and SAHA increased expression of *GADD45A *in two prostate cancer cell lines, PC3 and DU145. Induction was slight in 22Rv1, and the inhibitors were inefficacious in LNCaP (Figure 4C). In spite of the response to 5-aza-dC, bisulfite sequencing revealed the *GADD45A *promoter to be unmethylated in prostate cancer cell lines and prostate cancer tissues with low expression (Figure 4D).

## Discussion

The starting point of the present investigation was our previous observation that chromosome 8 alterations and LINE-1 hypomethylation concurred in many prostate cancers [8]. Such a relationship could be mechanistic in the sense that one alteration causes the other. For instance, chromosome 1 alterations may sometimes be a consequence of hypomethylation of CpG-rich satellites enriched in its juxtacentromeric heterochromatin [28]. Clearly, the fact that the highly significant association was not replicated in the present study makes mechanistic relationships of this kind unlikely. The major difference in the former study was a high proportion of metastatic and recurrent cancers, which exhibited LINE-1 hypomethylation regularly and chromosome 8 alterations often. Therefore, a likely explanation for the discrepancy is that LINE-1 hypomethylation and chromosome 8 alterations both contribute to prostate cancer progression, but are brought about by independent causes.

The fact that they concur in advanced cancers suggests a synergism between the two factors that could be exerted in two ways. Each alteration could target independent biological processes contributing to tumor progression, e.g. one might promote cell proliferation, while the other blocks apoptosis. Alternatively, both factors could target the same process(es). The expression profiling experiment in the present study was designed to distinguish between these alternatives and to identify relevant biological processes. The results clearly indicate that the two alterations act upon the same processes. Very few genes were significantly different between the tumors with or without LINE-1 hypomethylation or between those with or without chromosome 8 alterations, whereas interaction analysis identified several clearly circumscribed biological processes. One prominent process can be roughly categorized as suppression of immune responses, a second one as altered cytoskeletal organization and cell adhesion. Both processes are considered generally important in carcinogenesis, especially during tumor progression. Of note, these processes were identified by the interaction analysis as particularly important in the group of cancers with both alterations. The analysis of individual genes by quantitative real-time PCR revealed that many (e.g. *MX1*, *EPB41L3*), but not all (notably *BCCIP *and *TLR3*) of the genes significant in the interaction analysis were also overexpressed generally in prostate cancers compared to benign tissues.

More specifically, most individual genes differentially expressed in the immune-response GO groups relate to the activation of innate immune responses following viral infection. A key step in this response is the production of type I interferons by infected cells. These exert antiproliferative effects themselves and initiate adaptive immune responses. Antiproliferative and proapoptotic factors from T-cells, including IFNγ, then help to eliminate infected cells. The same sequence is thought to be involved in immunosurveillance against tumors caused by viruses and other carcinogens [29]. In particular, loss of responsiveness to IFNγ has previously been linked to prostate cancer progression [30,31]. This result is strengthened by our finding that genes associated with interferon responses are down-regulated in prostate cancer compared to normal tissue overall and even more so in a subgroup of cancers harboring molecular alterations typical of advanced stage tumors. Importantly, however, the genes identified as downregulated in our analysis include several that are involved in the earlier phase mediated by type I interferons, especially the prototypic type I interferon response genes *MX1 *and *MX2*. Of note, MX1 has a direct antiproliferative effect [32], as does GADD45α [33]. *IFNB1 *itself and *IRF3 *encoding a key transcription factor for its synthesis appeared downregulated in the group of prostate cancers with both alterations, too. In addition, expression of certain toll-like receptors including TLR2 and TLR3 that recognize 'alien' structures was diminished, at least in this subgroup. Downregulation of TLR3, which detects dsRNA of viral origin and aberrantly methylated endogenous RNAs [34] is apparent from a previous prostate cancer microarray study, but not was not followed up there [15]. Our findings thus suggest that the immune response to some prostate cancers may fail at at an early step because the cancers are not recognized. The finding that *TLR3 *down-regulation occurred in a subset of cancers and was associated with recurrence may mean that some cancers are more successful than others in escaping detection by the immune system.

Our findings are particularly intriguing when considered together with current knowledge on hereditary prostate cancer genes. The best characterized *HPC1 *gene encodes RNaseL, a protein involved in response to dsRNA produced during viral infections or certain other aberrant RNAs [35]. Another hereditary prostate cancer gene candidate is *MSR1 *at 8p23 likewise involved in innate immune responses [1]. Polymorphisms in *TLR *genes have also been implicated as predisposition factors [36,37]. Intriguingly, two genes (*BCCIP*, *HMG20B*) appeared in our analysis that interact with *BRCA2*, another, albeit weaker candidate for a hereditary prostate cancer gene [1]. *GADD45A *found downregulated in prostate cancers in our study is a well-established BRCA1 target. BRCA1 and IFNγ together act upon the RNaseL pathway [38].

The preponderance of immune response genes among hereditary prostate cancer genes has been interpreted as indicating a role for an aberrant immune response towards a viral agent in prostate carcinogenesis [1,35]. Obviously, our data are in line with that interpretation. Of note, the cancers displaying the most pronounced down-regulation of immune response-related genes harbored significant hypomethylation of LINE-1 retrotransposons and chromosome 8 alterations. Diminished methylation may facilitate LINE-1 reexpression [39]. LINE-1 hypomethylation in cancer parallels that of HERV endogenous retroviruses [39,40] and of ALU (SINE) sequences [40]. Activation of endogenous retroelements occurs in response to various types of genotoxic stress and may also take place during infection by exogenous viruses (reviewed in [41]). Indeed, processed HERV transcripts have been observed in prostate cancer [42]. HERV proteins are recognized as autoantigens in cancer and autoimmune diseases [41,43]. ALU transcripts influence the dsRNA-dependent protein kinase PKR, which regulates RNaseL activation, and TLRs [44]. Conceivably, therefore, what appears as an aberrant response to an exogenous viral agent in prostate carcinogenesis might reflect to some extent the activation of endogenous retroelements. Interestingly, the recent completion of the chromosome 8 sequence has highlighted an unusual concentration of genes involved in the regulation of the immune response on this particular chromosome [45]. Therefore, a speculative interpretation of our findings is that increased expression of endogenous retroelements in prostate cancer cells with hypomethylated genomes would contribute to the activation of immune responses, but is tolerated as a consequence of chromosome 8 alterations. We suggest this as a working hypothesis for further studies.

Similarly, our study implies that altered expression patterns of cytoskeletal and extracellular matrix proteins in prostate carcinoma are linked to alterations of chromosome 8 and LINE-1 hypomethylation. A novel finding in this context is the involvement of 4.1 proteins in prostate cancer. These proteins contain a phosphatidyl inositol phosphate-binding domain and connect a variety of transmembrane proteins to the actin cytoskeleton, thereby organizing cell polarity and motility. The two members of the family investigated here more closely are already implicated in other cancers. *EPB41L3 *was proposed as a potential tumor suppressor in lung cancer [25] and was shown to be down-regulated by promoter hypermethylation and allelic loss in renal cell carcinoma [26]. We report here that hypermethylation of *EPB41L3 *is also prevalent in prostate cancer. The 18p losses detected by CGH in 11/50 cases, could contribute to down-regulation. EHM2 encoded by *EPB41L4B *was identified as overexpressed in metastatic melanoma cells [46]. We show here that its overexpression is common in prostate cancer. This is the first explicit study on this gene, but our findings are in line with significant differences in previous microarray data [14,15]. Taken together, the results suggest a shift in the pattern of 4.1 proteins associated with prostate cancer progression whose biological and clinical implications deserve further investigation.

Altered expression of Fibulin-1, which interacts with laminins reported to be downregulated in several previous studies [14-17], was previously reported only in gynaecological cancers [47,48]. In these cancers, *FBLN1 *becomes upregulated during progression. In contrast and therefore surprisingly, in prostate cancer *FBLN1 *appears to become generally downregulated. The decreases in laminin and Fibulin-1 expression may be related to the dissolution of the basement membrane in prostate cancer tissues. Like the 4.1 proteins, Fibulin-1 influences cell motility and polarity. As overexpression and abnormal localization in breast cancer can lead to recognition of Fibulin-1 as an autoantigen [48], its decreased expression in prostate cancers may also lower their immunogenicity.

Promoter hypermethylation underlies *GADD45A *downregulation in breast cancer [27]. In prostate cancer altered methylation at a more distant, but unfortunately unspecified site in the gene has been reported [49]. We found the actual promoter unmethylated in prostate cancer tissues and cell lines. Induction of *GADD45A *by SAHA treatment in prostate cancer cell lines could mean that downregulation is associated with histone deacetylation and altered chromatin structure. The additive effect of 5-aza-dC treatment might reflect the methylation at the distant site [49].

## Conclusion

LINE-1 hypomethylation and chromosome 8 alterations are commonly associated in highly advanced prostate cancers. The present study suggests that both alterations do not cause each other. Rather, they appear to act as converging and even synergistic factors contributing to prostate cancer progression. Interaction analysis identified suppression of innate immune responses and cytoskeletal and extracellular matrix changes as common targets. This implies that candidate tumor genes on chromosome 8 as well as DNA hypomethylation should be considered for their influence on these processes in prostate cancer. Intriguingly, many hereditary prostate cancers genes are also involved in regulation of innate immune responses. The data stress the importance of altered cell adhesion and cytoskeletal organization in prostate cancer and specifically implicate changes in 4.1 protein expression in this process. The 4.1 protein encoding genes and several others newly identified here ought to be investigated for their usefulness in prostate cancer detection and classification through expression or methylation analyses.

## Methods

### Tissue samples

From a previous study of DNA methylation alterations in a series of 113 prostate carcinomas [9], a subset of 50 specimens (Table 1) was selected according to DNA availability, RNA quality and complete follow-up (median period 62 months). Cases with distant metastases at the time of surgery were excluded. Twelve cases had lymph node metastases. Benign tissues were taken from distant locations of cancer-carrying prostates as described [9]. The study was approved by the HHU medical faculty ethics committee.

### Cell lines

The prostate carcinoma cell lines LNCaP, 22RV1, PC3, and DU145 were cultured and treated with epigenetic inhibitors, 5-aza-2'-deoxycytidine (5-aza-dC; Sigma, Taufkirchen, Germany), suberoylanilide hydroxamic acid (SAHA, Biomol, Hamburg, Germany) as described [11]. 5-aza-dC was supplied at 2 μM every 24 h for 3 days and suberoylanilide hydroxamic acid at 2 μM for 2 days.

### DNA and RNA extraction

DNA and RNA were extracted from identical powdered tissues as described previously [9,11].

### Comparative genomic hybridization

CGH was performed as described [12], using the same aliquots as for DNA methylation analysis.

### RNA microarray analysis

High-quality total RNA (5 μg each) was converted to double-stranded cDNA before *in vitro *transcription with biotinylated dNTPs using the GeneChip^® ^Expression 3' Amplification One-Cycle Target Labeling Kit (Affymetrix, Santa Clara, USA). The resulting biotinylated cRNA was fragmented and hybridized to HG-U133A (Affymetrix) microarrays according to the manufacturer's specifications. After hybridization, the microarrays were washed, stained with streptavidin/phycoerythrin conjugate and biotinylated antibody, and scanned according to the manufacturer's recommendations using an Affymetrix GeneChip^® ^Scanner 3000. Images were processed using GeneChip^® ^Operating Software (GCOS, Version 1.3, Affymetrix) and total intensity normalization was applied by normalizing all arrays to an average signal level of 500 counts.

### Bioinformatic analysis of microarray data

#### Interaction analysis

For every gene separately, a multivariate linear model for predicting logarithmic gene expression was estimated. Two main effects for alteration of chromosome 8 and for hypomethylation of LINE-1 retrotransposons and an interaction effect for joint occurrence of these two factors were included in the model. Significance values for every gene and for the three effects, respectively, were obtained by applying the function *lm *for fitting linear models as implemented in the R programming language R [50].

#### GO group scoring

Two methods for scoring the significance of enrichment of a list of differentially expressed genes with genes belonging to a Gene Ontology (GO) group were applied, namely the *classic *and the *weight *algorithm [13]. In both algorithms, genes are first ranked according to a score that quantifies the amount of differential expression. Corresponding p-values are then adjusted for multiple testing according to the *false discovery rate (fdr) *method. All genes with adjusted p-values *p < 0.05 *are included in the *list of significant genes*. The significance of a GO term is then obtained by comparing the observed number of genes in the respective GO group that are members of this list with the expected number calculated in a model that assumes independence between the GO group and the list of significant genes. In the *classic *algorithm, this comparison is based on Fisher's exact test which is applied for each GO term independently. Due to the graph topology of the Gene Ontology, pairs of terms with parent-child relationships automatically receive similar p-values and thus appear simultaneously among the most significant GO terms. In the *weight *algorithm, genes annotated to a GO term receive weights based on the significance of neighboring GO terms [13]. This approach identifies local dependencies between related GO terms and highlights those terms that receive a more significant score than all their neighbors. The algorithms for GO group scoring were implemented in the R programming language [50]. The results were obtained using R version 2.3.0 and the libraries provided by the Bioconductor project, version 1.8 (released on April 27^th^, 2006).

### Quantitative RT-PCR

Quantitative real-time RT-PCR was performed using RNA from the identical tissue specimens on an ABI 7900 instrument using commercially available primers and probes specific for the respective mRNAs (Applied Biosystems, Weiterstadt, Germany). Each run was standardized using a dilution series of a strongly expressing cell line or normal tissue. Experimental variation for each sample was below 10%. All results were expressed relative to *TBP *used as a reference gene.

### DNA methylation analysis

Bisulfite sequencing DNA methylation analysis was performed as described [9,11] using the following novel primers for *EPB41L3 *DAL1fwd 5'-GTAATAGGGGG(T/C)GGGGGGAATAG-3', DAL1rev 5'-AACCCCCTC(A/G)CAATCCCCCACTC-3' for *GADD45A*: GADD45Afwd 5'-TTAGTGGTTGGTAGGTAGTGGTT-3', GADD45Arev 5'-CCTCCAAAATCATATTACAAACTAC-3'. Methylation-specific PCR was performed as described [9] using the novel primer pairs for *EPB41L3 *DAL1US 5'-TTTGTGTATTGTTGTTGAGGAGTG-3' and DAL1UAS 5'-CACAATCCCCCACTCCAAAAAACA-3' to detect unmethylated sequences or DAL1MS 5'-TTGCGTATCGTCGTCGTCGAGGACG-3' and DAL1MAS 5'-CGCAATCCCCCACTCCGAAAAACG-3' to detect methylated sequences at 61°C and 64°C annealing temperature, respectively.

## Competing interests

The author(s) declare that they have no competing interests.

## Authors' contributions

WAS, BW, and JR conceived the study. WAS and JR drafted the manuscript. VJ performed the CGH analysis evaluating it together with BW. CH, MJH, MY, SF, AW, and ARF carried out and evaluated the expression and methylation analyses with guidance by WAS. AA, TL, and JR performed the bioinformatic analyses. MM contributed the clinical data and according statistical evaluations. RE controlled the tissue sampling and histopathological data. All authors read and approved the final manuscript.

## Supplementary Material

Additional file 1 Table of genes significant in the 20 most significant GO groups. Genes annotated to the 20 most significant GO groups that are significantly differentially expressed (p < 0.01) between prostate cancer groups according to interaction analysis Click here for file

Additional file 2 Box plot representation of microarray analysis results for genes encoding markers of innate immune-response cell types. In each graph, the expression values (log-scale) for the respective genes are depicted for the cancers with neither hypomethylation of LINE-1 retrotransposons nor alteration of chromosome 8 (null), hypomethylation only (hypo), alteration of chromosome 8 only (chrom8) and both alterations (chrom8 AND hypo). The p-values refer to the result of the interaction analysis (see Methods for details). *KLRD1 *and *ITGA2 *gene products are characteristic of NK cells, *CPA3 *of mast cells, *CD163 *is relatively characteristic of macrophages (the more characteristic marker *CD68 *was not represented on the HGU133A microarray), *CD83 *and *ADAM19 *are typical for dendritic cells, and *ITGAM *encodes a marker of granulocytes. Click here for file

Additional file 3 Additional legend to figure 1. List of GO numbers and GO terms depicted in Fig. 1B and 1C Click here for file
