# Supplementary material for: Factor interaction analysis for chromosome 8 and DNA methylation alterations highlights innate immune response suppression and cytoskeletal changes in prostate cancer
Source: Mol Cancer. 2007 Feb 5;6:14. doi: 10.1186/1476-4598-6-14 (PMC1797054; doi:10.1186/1476-4598-6-14)
Supplement: Additional file 1 — Table of genes significant in the 20 most significant GO groups. Genes annotated to the 20 most significant GO groups that are significantly differentially expressed (p < 0.01) between prostate cancer groups according to interaction analysis [file 1476-4598-6-14-S1.doc]

**Supplementary table 1**:

| **GO:0006955 immune response** | | | | | |
| --- | --- | --- | --- | --- | --- |
| No | Affy ID | LL.id | Symbol.id | Gene name | raw p-value |
| 1 | 202086_at | 4599 | MX1 | myxovirus (influenza virus) resistance 1, interferon-inducible protein p78 (mouse) | 4.11E-05 |
| 2 | 213800_at | 3075 | CFH | complement factor H | 0.000181 |
| 3 | 205789_at | 912 | CD1D | CD1d molecule | 0.000854 |
| 4 | 203561_at | 2212 | FCGR2A | Fc fragment of IgG, low affinity IIa, receptor (CD32) | 0.000862 |
| 5 | 200986_at | 710 | SERPING1 | serpin peptidase inhibitor, clade G (C1 inhibitor), member 1, (angioedema, hereditary) | 0.001077 |
| 6 | 205270_s_at | 3937 | LCP2 | lymphocyte cytosolic protein 2 (SH2 domain containing leukocyte protein of 76kDa) | 0.001245 |
| 7 | 203104_at | 1436 | CSF1R | colony stimulating factor 1 receptor, formerly McDonough feline sarcoma viral (v-fms) oncogene homol... | 0.001301 |
| 8 | 204279_at | 5698 | PSMB9 | proteasome (prosome, macropain) subunit, beta type, 9 (large multifunctional peptidase 2) | 0.001343 |
| 9 | 204655_at | 6352 | CCL5 | chemokine (C-C motif) ligand 5 | 0.001589 |
| 10 | 209901_x_at | 199 | AIF1 | allograft inflammatory factor 1 | 0.001949 |
| 11 | 210629_x_at | 7940 | LST1 | leukocyte specific transcript 1 | 0.001952 |
| 12 | 205859_at | 9450 | LY86 | lymphocyte antigen 86 | 0.001968 |
| 13 | 208262_x_at | 4210 | MEFV | Mediterranean fever | 0.002091 |
| 14 | 204924_at | 7097 | TLR2 | toll-like receptor 2 | 0.002217 |
| 15 | 214511_x_at | 440607 | LOC440607 | NA | 0.002248 |
| 16 | 212067_s_at | 715 | C1R | complement component 1, r subcomponent | 0.0023 |
| 17 | 204158_s_at | 10312 | TCIRG1 | T-cell, immune regulator 1, ATPase, H+ transporting, lysosomal V0 subunit A3 | 0.002372 |
| 18 | 203854_at | 3426 | CFI | complement factor I | 0.002393 |
| 19 | 211795_s_at | 2533 | FYB | FYN binding protein (FYB-120/130) | 0.002648 |
| 20 | 210223_s_at | 3140 | MR1 | major histocompatibility complex, class I-related | 0.003039 |
| 21 | 216950_s_at | 2209 | FCGR1A | Fc fragment of IgG, high affinity Ia, receptor (CD64) | 0.004148 |
| 22 | 218638_s_at | 10417 | SPON2 | spondin 2, extracellular matrix protein | 0.004406 |
| 23 | 221087_s_at | 80833 | APOL3 | apolipoprotein L, 3 | 0.00456 |
| 24 | 205269_at | 3937 | LCP2 | lymphocyte cytosolic protein 2 (SH2 domain containing leukocyte protein of 76kDa) | 0.004964 |
| 25 | 205639_at | 313 | AOAH | acyloxyacyl hydrolase (neutrophil) | 0.005142 |
| 26 | 212758_s_at | 6935 | TCF8 | transcription factor 8 (represses interleukin 2 expression) | 0.005386 |
| 27 | 209813_x_at | 6983 | TRGV9 | T cell receptor gamma variable 9 | 0.005412 |
| 28 | 221601_s_at | 11009 | IL24 | interleukin 24 | 0.00545 |
| 29 | 209732_at | 9976 | CLEC2B | C-type lectin domain family 2, member B | 0.006334 |
| 30 | 213160_at | 1794 | DOCK2 | dedicator of cytokinesis 2 | 0.006466 |
| 31 | 209823_x_at | 3119 | HLA-DQB1 | major histocompatibility complex, class II, DQ beta 1 | 0.006689 |
| 32 | 203523_at | 4046 | LSP1 | lymphocyte-specific protein 1 | 0.006714 |
| 33 | 212998_x_at | 3119 | HLA-DQB1 | major histocompatibility complex, class II, DQ beta 1 | 0.006793 |
| 34 | 210972_x_at | 6955 | TRA@ | T cell receptor alpha locus | 0.006912 |
| 35 | 210140_at | 8530 | CST7 | cystatin F (leukocystatin) | 0.006948 |
| 36 | 204950_at | 22900 | CARD8 | caspase recruitment domain family, member 8 | 0.00708 |
| 37 | 205798_at | 3575 | IL7R | interleukin 7 receptor | 0.007273 |
| 38 | 204140_at | 8460 | TPST1 | tyrosylprotein sulfotransferase 1 | 0.007494 |
| 39 | 217369_at | 3500 | IGHG1 | immunoglobulin heavy constant gamma 1 (G1m marker) | 0.007524 |
| 40 | 211582_x_at | 7940 | LST1 | leukocyte specific transcript 1 | 0.008175 |
| 41 | 200904_at | 3133 | HLA-E | major histocompatibility complex, class I, E | 0.008298 |
| 42 | 222292_at | 958 | CD40 | CD40 molecule, TNF receptor superfamily member 5 | 0.00832 |
| 43 | 217767_at | 718 | C3 | complement component 3 | 0.008353 |
| 44 | 211991_s_at | 3113 | HLA-DPA1 | major histocompatibility complex, class II, DP alpha 1 | 0.008416 |
| 45 | 212587_s_at | 5788 | PTPRC | protein tyrosine phosphatase, receptor type, C | 0.008676 |
| 46 | 206978_at | 1231 | CCR2 | chemokine (C-C motif) receptor 2 | 0.008682 |
| 47 | 204122_at | 7305 | TYROBP | TYRO protein tyrosine kinase binding protein | 0.008874 |
| 48 | 204549_at | 9641 | IKBKE | inhibitor of kappa light polypeptide gene enhancer in B-cells, kinase epsilon | 0.009382 |
| 49 | 210029_at | 3620 | INDO | indoleamine-pyrrole 2,3 dioxygenase | 0.009383 |
| 50 | 210992_x_at | 9103 | FCGR2C | NA | 0.009499 |
| 51 | 220491_at | 57817 | HAMP | hepcidin antimicrobial peptide | 0.009548 |
| 52 | 200670_at | 7494 | XBP1 | X-box binding protein 1 | 0.009589 |
| 53 | 208747_s_at | 716 | C1S | complement component 1, s subcomponent | 0.009645 |
| 54 | 213293_s_at | 10346 | TRIM22 | tripartite motif-containing 22 | 0.009725 |
| 55 | 213475_s_at | 3683 | ITGAL | integrin, alpha L (antigen CD11A (p180), lymphocyte function-associated antigen 1; alpha polypeptide... | 0.009736 |
| 56 | 205831_at | 914 | CD2 | CD2 molecule | 0.009828 |
| 57 | 202748_at | 2634 | GBP2 | guanylate binding protein 2, interferon-inducible | 0.009997 |
| **GO:0009607 response to biotic stimulus** | | | | | |
| 1 | 202086_at | 4599 | MX1 | myxovirus (influenza virus) resistance 1, interferon-inducible protein p78 (mouse) | 4.11E-05 |
| 2 | 213800_at | 3075 | CFH | complement factor H | 0.000181 |
| 3 | 205789_at | 912 | CD1D | CD1d molecule | 0.000854 |
| 4 | 203561_at | 2212 | FCGR2A | Fc fragment of IgG, low affinity IIa, receptor (CD32) | 0.000862 |
| 5 | 200986_at | 710 | SERPING1 | serpin peptidase inhibitor, clade G (C1 inhibitor), member 1, (angioedema, hereditary) | 0.001077 |
| 6 | 205270_s_at | 3937 | LCP2 | lymphocyte cytosolic protein 2 (SH2 domain containing leukocyte protein of 76kDa) | 0.001245 |
| 7 | 203104_at | 1436 | CSF1R | colony stimulating factor 1 receptor, formerly McDonough feline sarcoma viral (v-fms) oncogene homol... | 0.001301 |
| 8 | 204279_at | 5698 | PSMB9 | proteasome (prosome, macropain) subunit, beta type, 9 (large multifunctional peptidase 2) | 0.001343 |
| 9 | 204655_at | 6352 | CCL5 | chemokine (C-C motif) ligand 5 | 0.001589 |
| 10 | 209901_x_at | 199 | AIF1 | allograft inflammatory factor 1 | 0.001949 |
| 11 | 210629_x_at | 7940 | LST1 | leukocyte specific transcript 1 | 0.001952 |
| 12 | 205859_at | 9450 | LY86 | lymphocyte antigen 86 | 0.001968 |
| 13 | 208262_x_at | 4210 | MEFV | Mediterranean fever | 0.002091 |
| 14 | 204924_at | 7097 | TLR2 | toll-like receptor 2 | 0.002217 |
| 15 | 214511_x_at | 440607 | LOC440607 | NA | 0.002248 |
| 16 | 212067_s_at | 715 | C1R | complement component 1, r subcomponent | 0.0023 |
| 17 | 204158_s_at | 10312 | TCIRG1 | T-cell, immune regulator 1, ATPase, H+ transporting, lysosomal V0 subunit A3 | 0.002372 |
| 18 | 203854_at | 3426 | CFI | complement factor I | 0.002393 |
| 19 | 211795_s_at | 2533 | FYB | FYN binding protein (FYB-120/130) | 0.002648 |
| 20 | 210223_s_at | 3140 | MR1 | major histocompatibility complex, class I-related | 0.003039 |
| 21 | 202708_s_at | 8349 | HIST2H2BE | histone 2, H2be | 0.003981 |
| 22 | 216950_s_at | 2209 | FCGR1A | Fc fragment of IgG, high affinity Ia, receptor (CD64) | 0.004148 |
| 23 | 218638_s_at | 10417 | SPON2 | spondin 2, extracellular matrix protein | 0.004406 |
| 24 | 221087_s_at | 80833 | APOL3 | apolipoprotein L, 3 | 0.00456 |
| 25 | 205269_at | 3937 | LCP2 | lymphocyte cytosolic protein 2 (SH2 domain containing leukocyte protein of 76kDa) | 0.004964 |
| 26 | 211563_s_at | 8725 | C19orf2 | chromosome 19 open reading frame 2 | 0.005081 |
| 27 | 205639_at | 313 | AOAH | acyloxyacyl hydrolase (neutrophil) | 0.005142 |
| 28 | 204118_at | 962 | CD48 | CD48 molecule | 0.005245 |
| 29 | 212758_s_at | 6935 | TCF8 | transcription factor 8 (represses interleukin 2 expression) | 0.005386 |
| 30 | 209813_x_at | 6983 | TRGV9 | T cell receptor gamma variable 9 | 0.005412 |
| 31 | 221601_s_at | 11009 | IL24 | interleukin 24 | 0.00545 |
| 32 | 202621_at | 3661 | IRF3 | interferon regulatory factor 3 | 0.005753 |
| 33 | 209732_at | 9976 | CLEC2B | C-type lectin domain family 2, member B | 0.006334 |
| 34 | 213160_at | 1794 | DOCK2 | dedicator of cytokinesis 2 | 0.006466 |
| 35 | 209823_x_at | 3119 | HLA-DQB1 | major histocompatibility complex, class II, DQ beta 1 | 0.006689 |
| 36 | 203523_at | 4046 | LSP1 | lymphocyte-specific protein 1 | 0.006714 |
| 37 | 212998_x_at | 3119 | HLA-DQB1 | major histocompatibility complex, class II, DQ beta 1 | 0.006793 |
| 38 | 210972_x_at | 6955 | TRA@ | T cell receptor alpha locus | 0.006912 |
| 39 | 210140_at | 8530 | CST7 | cystatin F (leukocystatin) | 0.006948 |
| 40 | 204950_at | 22900 | CARD8 | caspase recruitment domain family, member 8 | 0.00708 |
| 41 | 205798_at | 3575 | IL7R | interleukin 7 receptor | 0.007273 |
| 42 | 204140_at | 8460 | TPST1 | tyrosylprotein sulfotransferase 1 | 0.007494 |
| 43 | 217369_at | 3500 | IGHG1 | immunoglobulin heavy constant gamma 1 (G1m marker) | 0.007524 |
| 44 | 201850_at | 822 | CAPG | capping protein (actin filament), gelsolin-like | 0.007525 |
| 45 | 211582_x_at | 7940 | LST1 | leukocyte specific transcript 1 | 0.008175 |
| 46 | 200904_at | 3133 | HLA-E | major histocompatibility complex, class I, E | 0.008298 |
| 47 | 222292_at | 958 | CD40 | CD40 molecule, TNF receptor superfamily member 5 | 0.00832 |
| 48 | 217767_at | 718 | C3 | complement component 3 | 0.008353 |
| 49 | 211991_s_at | 3113 | HLA-DPA1 | major histocompatibility complex, class II, DP alpha 1 | 0.008416 |
| 50 | 212587_s_at | 5788 | PTPRC | protein tyrosine phosphatase, receptor type, C | 0.008676 |
| 51 | 206978_at | 1231 | CCR2 | chemokine (C-C motif) receptor 2 | 0.008682 |
| 52 | 204122_at | 7305 | TYROBP | TYRO protein tyrosine kinase binding protein | 0.008874 |
| 53 | 204549_at | 9641 | IKBKE | inhibitor of kappa light polypeptide gene enhancer in B-cells, kinase epsilon | 0.009382 |
| 54 | 210029_at | 3620 | INDO | indoleamine-pyrrole 2,3 dioxygenase | 0.009383 |
| 55 | 210992_x_at | 9103 | FCGR2C | NA | 0.009499 |
| 56 | 220491_at | 57817 | HAMP | hepcidin antimicrobial peptide | 0.009548 |
| 57 | 200670_at | 7494 | XBP1 | X-box binding protein 1 | 0.009589 |
| 58 | 208747_s_at | 716 | C1S | complement component 1, s subcomponent | 0.009645 |
| 59 | 213293_s_at | 10346 | TRIM22 | tripartite motif-containing 22 | 0.009725 |
| 60 | 213475_s_at | 3683 | ITGAL | integrin, alpha L (antigen CD11A (p180), lymphocyte function-associated antigen 1; alpha polypeptide... | 0.009736 |
| 61 | 205831_at | 914 | CD2 | CD2 molecule | 0.009828 |
| 62 | 206082_at | 10866 | HCP5 | HLA complex P5 | 0.009992 |
| 63 | 202748_at | 2634 | GBP2 | guanylate binding protein 2, interferon-inducible | 0.009997 |
| **GO:0030865 cortical cytoskeleton organization and biogenesis** | | | | | |
| 1 | 201718_s_at | 2037 | EPB41L2 | erythrocyte membrane protein band 4.1-like 2 | 0.000112 |
| 2 | 212339_at | 2036 | EPB41L1 | erythrocyte membrane protein band 4.1-like 1 | 0.003884 |
| 3 | 202796_at | 11346 | SYNPO | synaptopodin | 0.004357 |
| 4 | 211776_s_at | 23136 | EPB41L3 | erythrocyte membrane protein band 4.1-like 3 | 0.004912 |
| 5 | 206710_s_at | 23136 | EPB41L3 | erythrocyte membrane protein band 4.1-like 3 | 0.007327 |
| **GO:0001766 lipid raft polarization** | | | | | |
| 1 | 204777_s_at | 4118 | MAL | mal, T-cell differentiation protein | 0.00687 |
| 2 | 205831_at | 914 | CD2 | CD2 molecule | 0.00983 |
| **GO:0006956 complement activation** | | | | | |
| 1 | 213800_at | 3075 | CFH | complement factor H | 0.000181 |
| 2 | 200986_at | 710 | SERPING1 | serpin peptidase inhibitor, clade G (C1 inhibitor), member 1, (angioedema, hereditary) | 0.001077 |
| 3 | 212067_s_at | 715 | C1R | complement component 1, r subcomponent | 0.0023 |
| 4 | 203854_at | 3426 | CFI | complement factor I | 0.002393 |
| 5 | 217767_at | 718 | C3 | complement component 3 | 0.008353 |
| 6 | 208747_s_at | 716 | C1S | complement component 1, s subcomponent | 0.009645 |
| **GO:0007155 cell adhesion** | | | | | |
| 1 | 201792_at | 165 | AEBP1 | AE binding protein 1 | 0.000363 |
| 2 | 213519_s_at | 3908 | LAMA2 | laminin, alpha 2 (merosin, congenital muscular dystrophy) | 0.000413 |
| 3 | 211651_s_at | 3912 | LAMB1 | laminin, beta 1 | 0.00049 |
| 4 | 204358_s_at | 23768 | FLRT2 | fibronectin leucine rich transmembrane protein 2 | 0.000524 |
| 5 | 216840_s_at | 3908 | LAMA2 | laminin, alpha 2 (merosin, congenital muscular dystrophy) | 0.000582 |
| 6 | 212713_at | 4239 | MFAP4 | microfibrillar-associated protein 4 | 0.000974 |
| 7 | 211203_s_at | 1272 | CNTN1 | contactin 1 | 0.001329 |
| 8 | 204655_at | 6352 | CCL5 | chemokine (C-C motif) ligand 5 | 0.001589 |
| 9 | 211571_s_at | 1462 | CSPG2 | chondroitin sulfate proteoglycan 2 (versican) | 0.001625 |
| 10 | 201506_at | 7045 | TGFBI | transforming growth factor, beta-induced, 68kDa | 0.002947 |
| 11 | 203045_at | 4814 | NINJ1 | ninjurin 1 | 0.003062 |
| 12 | 206470_at | 10154 | PLXNC1 | plexin C1 | 0.003087 |
| 13 | 205168_at | 4921 | DDR2 | discoidin domain receptor family, member 2 | 0.003176 |
| 14 | 207191_s_at | 3671 | ISLR | immunoglobulin superfamily containing leucine-rich repeat | 0.003861 |
| 15 | 218084_x_at | 53827 | FXYD5 | FXYD domain containing ion transport regulator 5 | 0.004237 |
| 16 | 218638_s_at | 10417 | SPON2 | spondin 2, extracellular matrix protein | 0.004406 |
| 17 | 202363_at | 6695 | SPOCK1 | sparc/osteonectin, cwcv and kazal-like domains proteoglycan (testican) 1 | 0.004487 |
| 18 | 214265_at | 8516 | ITGA8 | integrin, alpha 8 | 0.004747 |
| 19 | 205786_s_at | 3684 | ITGAM | integrin, alpha M (complement component 3 receptor 3 subunit) | 0.004786 |
| 20 | 206580_s_at | 30008 | EFEMP2 | EGF-containing fibulin-like extracellular matrix protein 2 | 0.004787 |
| 21 | 205116_at | 3908 | LAMA2 | laminin, alpha 2 (merosin, congenital muscular dystrophy) | 0.004939 |
| 22 | 213993_at | 10418 | SPON1 | spondin 1, extracellular matrix protein | 0.004952 |
| 23 | 215646_s_at | 1462 | CSPG2 | chondroitin sulfate proteoglycan 2 (versican) | 0.00501 |
| 24 | 211042_x_at | 4162 | MCAM | melanoma cell adhesion molecule | 0.005145 |
| 25 | 215775_at | 7057 | THBS1 | thrombospondin 1 | 0.005272 |
| 26 | 204359_at | 23768 | FLRT2 | fibronectin leucine rich transmembrane protein 2 | 0.005371 |
| 27 | 204620_s_at | 1462 | CSPG2 | chondroitin sulfate proteoglycan 2 (versican) | 0.005767 |
| 28 | 219213_at | 58494 | JAM2 | junctional adhesion molecule 2 | 0.007047 |
| 29 | 211432_s_at | 7301 | TYRO3 | TYRO3 protein tyrosine kinase | 0.007271 |
| 30 | 201505_at | 3912 | LAMB1 | laminin, beta 1 | 0.007341 |
| 31 | 204345_at | 1307 | COL16A1 | collagen, type XVI, alpha 1 | 0.008068 |
| 32 | 205884_at | 3676 | ITGA4 | integrin, alpha 4 (antigen CD49D, alpha 4 subunit of VLA-4 receptor) | 0.008632 |
| 33 | 209356_x_at | 30008 | EFEMP2 | EGF-containing fibulin-like extracellular matrix protein 2 | 0.008735 |
| 34 | 202638_s_at | 3383 | ICAM1 | intercellular adhesion molecule 1 (CD54), human rhinovirus receptor | 0.00961 |
| 35 | 213475_s_at | 3683 | ITGAL | integrin, alpha L (antigen CD11A (p180), lymphocyte function-associated antigen 1; alpha polypeptide... | 0.009736 |
| 36 | 205831_at | 914 | CD2 | CD2 molecule | 0.009828 |
| **GO:0009967 positive regulation of signal transduction** | | | | | |
| 1 | 209365_s_at | 1893 | ECM1 | extracellular matrix protein 1 | 0.00134 |
| 2 | 58994_at | 54862 | CC2D1A | coiled-coil and C2 domain containing 1A | 0.00176 |
| 3 | 221087_s_at | 80833 | APOL3 | apolipoprotein L, 3 | 0.00456 |
| 4 | 210512_s_at | 7422 | VEGF | vascular endothelial growth factor | 0.00554 |
| 5 | 204037_at | 1902 | EDG2 | endothelial differentiation, lysophosphatidic acid G-protein-coupled receptor, 2 | 0.00616 |
| 6 | 206170_at | 154 | ADRB2 | adrenergic, beta-2-, receptor, surface | 0.00811 |
| 7 | 203236_s_at | 3965 | LGALS9 | lectin, galactoside-binding, soluble, 9 (galectin 9) | 0.00815 |
| 8 | 222292_at | 958 | CD40 | CD40 molecule, TNF receptor superfamily member 5 | 0.00832 |
| 9 | 204036_at | 1902 | EDG2 | endothelial differentiation, lysophosphatidic acid G-protein-coupled receptor, 2 | 0.00863 |
| 10 | 212587_s_at | 5788 | PTPRC | protein tyrosine phosphatase, receptor type, C | 0.00868 |
| 11 | 204549_at | 9641 | IKBKE | inhibitor of kappa light polypeptide gene enhancer in B-cells, kinase epsilon | 0.00938 |
| **GO:0009611 response to wounding** | | | | | |
| 1 | 209844_at | 10481 | HOXB13 | homeobox B13 | 0.000969 |
| 2 | 200986_at | 710 | SERPING1 | serpin peptidase inhibitor, clade G (C1 inhibitor), member 1, (angioedema, hereditary) | 0.001077 |
| 3 | 204655_at | 6352 | CCL5 | chemokine (C-C motif) ligand 5 | 0.001589 |
| 4 | 209901_x_at | 199 | AIF1 | allograft inflammatory factor 1 | 0.001949 |
| 5 | 205859_at | 9450 | LY86 | lymphocyte antigen 86 | 0.001968 |
| 6 | 208262_x_at | 4210 | MEFV | Mediterranean fever | 0.002091 |
| 7 | 204924_at | 7097 | TLR2 | toll-like receptor 2 | 0.002217 |
| 8 | 204158_s_at | 10312 | TCIRG1 | T-cell, immune regulator 1, ATPase, H+ transporting, lysosomal V0 subunit A3 | 0.002372 |
| 9 | 203045_at | 4814 | NINJ1 | ninjurin 1 | 0.003062 |
| 10 | 221087_s_at | 80833 | APOL3 | apolipoprotein L, 3 | 0.00456 |
| 11 | 206580_s_at | 30008 | EFEMP2 | EGF-containing fibulin-like extracellular matrix protein 2 | 0.004787 |
| 12 | 205639_at | 313 | AOAH | acyloxyacyl hydrolase (neutrophil) | 0.005142 |
| 13 | 215775_at | 7057 | THBS1 | thrombospondin 1 | 0.005272 |
| 14 | 209813_x_at | 6983 | TRGV9 | T cell receptor gamma variable 9 | 0.005412 |
| 15 | 213160_at | 1794 | DOCK2 | dedicator of cytokinesis 2 | 0.006466 |
| 16 | 203523_at | 4046 | LSP1 | lymphocyte-specific protein 1 | 0.006714 |
| 17 | 210972_x_at | 6955 | TRA@ | T cell receptor alpha locus | 0.006912 |
| 18 | 204140_at | 8460 | TPST1 | tyrosylprotein sulfotransferase 1 | 0.007494 |
| 19 | 210664_s_at | 7035 | TFPI | tissue factor pathway inhibitor (lipoprotein-associated coagulation inhibitor) | 0.008215 |
| 20 | 222292_at | 958 | CD40 | CD40 molecule, TNF receptor superfamily member 5 | 0.00832 |
| 21 | 217767_at | 718 | C3 | complement component 3 | 0.008353 |
| 22 | 206978_at | 1231 | CCR2 | chemokine (C-C motif) receptor 2 | 0.008682 |
| 23 | 209356_x_at | 30008 | EFEMP2 | EGF-containing fibulin-like extracellular matrix protein 2 | 0.008735 |
| 24 | 204122_at | 7305 | TYROBP | TYRO protein tyrosine kinase binding protein | 0.008874 |
| 25 | 213475_s_at | 3683 | ITGAL | integrin, alpha L (antigen CD11A (p180), lymphocyte function-associated antigen 1; alpha polypeptide... | 0.009736 |
| **GO:0045995 regulation of embryonic development** | | | | | |
| 1 | 213519_s_at | 3908 | LAMA2 | laminin, alpha 2 (merosin, congenital muscular dystrophy) | 0.000413 |
| 2 | 216840_s_at | 3908 | LAMA2 | laminin, alpha 2 (merosin, congenital muscular dystrophy) | 0.000582 |
| 3 | 205116_at | 3908 | LAMA2 | laminin, alpha 2 (merosin, congenital muscular dystrophy) | 0.004939 |
| **GO:0042135 neurotransmitter catabolism** | | | | | |
| 1 | 212741_at | 4128 | MAOA | monoamine oxidase A | 0.00446 |
| 2 | 204388_s_at | 4128 | MAOA | monoamine oxidase A | 0.00573 |
| 3 | 204389_at | 4128 | MAOA | monoamine oxidase A | 0.00573 |
| **GO:0043122 regulation of I-kappaB kinase/NF-kappaB cascade** | | | | | |
| 1 | 209365_s_at | 1893 | ECM1 | extracellular matrix protein 1 | 0.00134 |
| 2 | 58994_at | 54862 | CC2D1A | coiled-coil and C2 domain containing 1A | 0.00176 |
| 3 | 221087_s_at | 80833 | APOL3 | apolipoprotein L, 3 | 0.00456 |
| 4 | 204037_at | 1902 | EDG2 | endothelial differentiation, lysophosphatidic acid G-protein-coupled receptor, 2 | 0.00616 |
| 5 | 204950_at | 22900 | CARD8 | caspase recruitment domain family, member 8 | 0.00708 |
| 6 | 203236_s_at | 3965 | LGALS9 | lectin, galactoside-binding, soluble, 9 (galectin 9) | 0.00815 |
| 7 | 222292_at | 958 | CD40 | CD40 molecule, TNF receptor superfamily member 5 | 0.00832 |
| 8 | 204036_at | 1902 | EDG2 | endothelial differentiation, lysophosphatidic acid G-protein-coupled receptor, 2 | 0.00863 |
| 9 | 204549_at | 9641 | IKBKE | inhibitor of kappa light polypeptide gene enhancer in B-cells, kinase epsilon | 0.00938 |
| **GO:0006952 defense response** | | | | | |
| 1 | 202086_at | 4599 | MX1 | myxovirus (influenza virus) resistance 1, interferon-inducible protein p78 (mouse) | 4.11E-05 |
| 2 | 213800_at | 3075 | CFH | complement factor H | 0.000181 |
| 3 | 205789_at | 912 | CD1D | CD1d molecule | 0.000854 |
| 4 | 203561_at | 2212 | FCGR2A | Fc fragment of IgG, low affinity IIa, receptor (CD32) | 0.000862 |
| 5 | 200986_at | 710 | SERPING1 | serpin peptidase inhibitor, clade G (C1 inhibitor), member 1, (angioedema, hereditary) | 0.001077 |
| 6 | 205270_s_at | 3937 | LCP2 | lymphocyte cytosolic protein 2 (SH2 domain containing leukocyte protein of 76kDa) | 0.001245 |
| 7 | 203104_at | 1436 | CSF1R | colony stimulating factor 1 receptor, formerly McDonough feline sarcoma viral (v-fms) oncogene homol... | 0.001301 |
| 8 | 204279_at | 5698 | PSMB9 | proteasome (prosome, macropain) subunit, beta type, 9 (large multifunctional peptidase 2) | 0.001343 |
| 9 | 204655_at | 6352 | CCL5 | chemokine (C-C motif) ligand 5 | 0.001589 |
| 10 | 209901_x_at | 199 | AIF1 | allograft inflammatory factor 1 | 0.001949 |
| 11 | 210629_x_at | 7940 | LST1 | leukocyte specific transcript 1 | 0.001952 |
| 12 | 205859_at | 9450 | LY86 | lymphocyte antigen 86 | 0.001968 |
| 13 | 208262_x_at | 4210 | MEFV | Mediterranean fever | 0.002091 |
| 14 | 204924_at | 7097 | TLR2 | toll-like receptor 2 | 0.002217 |
| 15 | 214511_x_at | 440607 | LOC440607 | NA | 0.002248 |
| 16 | 212067_s_at | 715 | C1R | complement component 1, r subcomponent | 0.0023 |
| 17 | 204158_s_at | 10312 | TCIRG1 | T-cell, immune regulator 1, ATPase, H+ transporting, lysosomal V0 subunit A3 | 0.002372 |
| 18 | 203854_at | 3426 | CFI | complement factor I | 0.002393 |
| 19 | 211795_s_at | 2533 | FYB | FYN binding protein (FYB-120/130) | 0.002648 |
| 20 | 210223_s_at | 3140 | MR1 | major histocompatibility complex, class I-related | 0.003039 |
| 21 | 202708_s_at | 8349 | HIST2H2BE | histone 2, H2be | 0.003981 |
| 22 | 216950_s_at | 2209 | FCGR1A | Fc fragment of IgG, high affinity Ia, receptor (CD64) | 0.004148 |
| 23 | 218638_s_at | 10417 | SPON2 | spondin 2, extracellular matrix protein | 0.004406 |
| 24 | 221087_s_at | 80833 | APOL3 | apolipoprotein L, 3 | 0.00456 |
| 25 | 205269_at | 3937 | LCP2 | lymphocyte cytosolic protein 2 (SH2 domain containing leukocyte protein of 76kDa) | 0.004964 |
| 26 | 205639_at | 313 | AOAH | acyloxyacyl hydrolase (neutrophil) | 0.005142 |
| 27 | 204118_at | 962 | CD48 | CD48 molecule | 0.005245 |
| 28 | 212758_s_at | 6935 | TCF8 | transcription factor 8 (represses interleukin 2 expression) | 0.005386 |
| 29 | 209813_x_at | 6983 | TRGV9 | T cell receptor gamma variable 9 | 0.005412 |
| 30 | 221601_s_at | 11009 | IL24 | interleukin 24 | 0.00545 |
| 31 | 209732_at | 9976 | CLEC2B | C-type lectin domain family 2, member B | 0.006334 |
| 32 | 213160_at | 1794 | DOCK2 | dedicator of cytokinesis 2 | 0.006466 |
| 33 | 209823_x_at | 3119 | HLA-DQB1 | major histocompatibility complex, class II, DQ beta 1 | 0.006689 |
| 34 | 203523_at | 4046 | LSP1 | lymphocyte-specific protein 1 | 0.006714 |
| 35 | 212998_x_at | 3119 | HLA-DQB1 | major histocompatibility complex, class II, DQ beta 1 | 0.006793 |
| 36 | 210972_x_at | 6955 | TRA@ | T cell receptor alpha locus | 0.006912 |
| 37 | 210140_at | 8530 | CST7 | cystatin F (leukocystatin) | 0.006948 |
| 38 | 204950_at | 22900 | CARD8 | caspase recruitment domain family, member 8 | 0.00708 |
| 39 | 205798_at | 3575 | IL7R | interleukin 7 receptor | 0.007273 |
| 40 | 204140_at | 8460 | TPST1 | tyrosylprotein sulfotransferase 1 | 0.007494 |
| 41 | 217369_at | 3500 | IGHG1 | immunoglobulin heavy constant gamma 1 (G1m marker) | 0.007524 |
| 42 | 211582_x_at | 7940 | LST1 | leukocyte specific transcript 1 | 0.008175 |
| 43 | 200904_at | 3133 | HLA-E | major histocompatibility complex, class I, E | 0.008298 |
| 44 | 222292_at | 958 | CD40 | CD40 molecule, TNF receptor superfamily member 5 | 0.00832 |
| 45 | 217767_at | 718 | C3 | complement component 3 | 0.008353 |
| 46 | 211991_s_at | 3113 | HLA-DPA1 | major histocompatibility complex, class II, DP alpha 1 | 0.008416 |
| 47 | 212587_s_at | 5788 | PTPRC | protein tyrosine phosphatase, receptor type, C | 0.008676 |
| 48 | 206978_at | 1231 | CCR2 | chemokine (C-C motif) receptor 2 | 0.008682 |
| 49 | 204122_at | 7305 | TYROBP | TYRO protein tyrosine kinase binding protein | 0.008874 |
| 50 | 204549_at | 9641 | IKBKE | inhibitor of kappa light polypeptide gene enhancer in B-cells, kinase epsilon | 0.009382 |
| 51 | 210029_at | 3620 | INDO | indoleamine-pyrrole 2,3 dioxygenase | 0.009383 |
| 52 | 210992_x_at | 9103 | FCGR2C | NA | 0.009499 |
| 53 | 220491_at | 57817 | HAMP | hepcidin antimicrobial peptide | 0.009548 |
| 54 | 200670_at | 7494 | XBP1 | X-box binding protein 1 | 0.009589 |
| 55 | 208747_s_at | 716 | C1S | complement component 1, s subcomponent | 0.009645 |
| 56 | 213293_s_at | 10346 | TRIM22 | tripartite motif-containing 22 | 0.009725 |
| 57 | 213475_s_at | 3683 | ITGAL | integrin, alpha L (antigen CD11A (p180), lymphocyte function-associated antigen 1; alpha polypeptide... | 0.009736 |
| 58 | 205831_at | 914 | CD2 | CD2 molecule | 0.009828 |
| 59 | 206082_at | 10866 | HCP5 | HLA complex P5 | 0.009992 |
| 60 | 202748_at | 2634 | GBP2 | guanylate binding protein 2, interferon-inducible | 0.009997 |
| **GO:0009190 cyclic nucleotide biosynthesis** | | | | | |
| 1 | 209320_at | 109 | ADCY3 | adenylate cyclase 3 | 0.000645 |
| 2 | 221942_s_at | 2982 | GUCY1A3 | guanylate cyclase 1, soluble, alpha 3 | 0.001159 |
| 3 | 209321_s_at | 109 | ADCY3 | adenylate cyclase 3 | 0.00231 |
| 4 | 213245_at | 107 | ADCY1 | adenylate cyclase 1 (brain) | 0.007323 |
| **GO:0050678 regulation of epithelial cell proliferation** | | | | | |
| 1 | 211651_s_at | 3912 | LAMB1 | laminin, beta 1 | 0.00049 |
| 2 | 201505_at | 3912 | LAMB1 | laminin, beta 1 | 0.00734 |
| 3 | 204197_s_at | 864 | RUNX3 | runt-related transcription factor 3 | 0.00905 |
| **GO:0008354 germ cell migration** | | | | | |
| 1 | 209147_s_at | 8611 | PPAP2A | phosphatidic acid phosphatase type 2A | 0.00214 |
| 2 | 210946_at | 8611 | PPAP2A | phosphatidic acid phosphatase type 2A | 0.00229 |
| **GO:0009613 response to pest, pathogen or parasite** | | | | | |
| 1 | 202086_at | 4599 | MX1 | myxovirus (influenza virus) resistance 1, interferon-inducible protein p78 (mouse) | 4.11E-05 |
| 2 | 213800_at | 3075 | CFH | complement factor H | 0.000181 |
| 3 | 200986_at | 710 | SERPING1 | serpin peptidase inhibitor, clade G (C1 inhibitor), member 1, (angioedema, hereditary) | 0.001077 |
| 4 | 203104_at | 1436 | CSF1R | colony stimulating factor 1 receptor, formerly McDonough feline sarcoma viral (v-fms) oncogene homol... | 0.001301 |
| 5 | 204655_at | 6352 | CCL5 | chemokine (C-C motif) ligand 5 | 0.001589 |
| 6 | 209901_x_at | 199 | AIF1 | allograft inflammatory factor 1 | 0.001949 |
| 7 | 205859_at | 9450 | LY86 | lymphocyte antigen 86 | 0.001968 |
| 8 | 208262_x_at | 4210 | MEFV | Mediterranean fever | 0.002091 |
| 9 | 204924_at | 7097 | TLR2 | toll-like receptor 2 | 0.002217 |
| 10 | 212067_s_at | 715 | C1R | complement component 1, r subcomponent | 0.0023 |
| 11 | 204158_s_at | 10312 | TCIRG1 | T-cell, immune regulator 1, ATPase, H+ transporting, lysosomal V0 subunit A3 | 0.002372 |
| 12 | 203854_at | 3426 | CFI | complement factor I | 0.002393 |
| 13 | 202708_s_at | 8349 | HIST2H2BE | histone 2, H2be | 0.003981 |
| 14 | 221087_s_at | 80833 | APOL3 | apolipoprotein L, 3 | 0.00456 |
| 15 | 211563_s_at | 8725 | C19orf2 | chromosome 19 open reading frame 2 | 0.005081 |
| 16 | 205639_at | 313 | AOAH | acyloxyacyl hydrolase (neutrophil) | 0.005142 |
| 17 | 209813_x_at | 6983 | TRGV9 | T cell receptor gamma variable 9 | 0.005412 |
| 18 | 202621_at | 3661 | IRF3 | interferon regulatory factor 3 | 0.005753 |
| 19 | 209732_at | 9976 | CLEC2B | C-type lectin domain family 2, member B | 0.006334 |
| 20 | 213160_at | 1794 | DOCK2 | dedicator of cytokinesis 2 | 0.006466 |
| 21 | 203523_at | 4046 | LSP1 | lymphocyte-specific protein 1 | 0.006714 |
| 22 | 210972_x_at | 6955 | TRA@ | T cell receptor alpha locus | 0.006912 |
| 23 | 205798_at | 3575 | IL7R | interleukin 7 receptor | 0.007273 |
| 24 | 204140_at | 8460 | TPST1 | tyrosylprotein sulfotransferase 1 | 0.007494 |
| 25 | 201850_at | 822 | CAPG | capping protein (actin filament), gelsolin-like | 0.007525 |
| 26 | 222292_at | 958 | CD40 | CD40 molecule, TNF receptor superfamily member 5 | 0.00832 |
| 27 | 217767_at | 718 | C3 | complement component 3 | 0.008353 |
| 28 | 212587_s_at | 5788 | PTPRC | protein tyrosine phosphatase, receptor type, C | 0.008676 |
| 29 | 206978_at | 1231 | CCR2 | chemokine (C-C motif) receptor 2 | 0.008682 |
| 30 | 204122_at | 7305 | TYROBP | TYRO protein tyrosine kinase binding protein | 0.008874 |
| 31 | 220491_at | 57817 | HAMP | hepcidin antimicrobial peptide | 0.009548 |
| 32 | 208747_s_at | 716 | C1S | complement component 1, s subcomponent | 0.009645 |
| 33 | 213293_s_at | 10346 | TRIM22 | tripartite motif-containing 22 | 0.009725 |
| 34 | 213475_s_at | 3683 | ITGAL | integrin, alpha L (antigen CD11A (p180), lymphocyte function-associated antigen 1; alpha polypeptide... | 0.009736 |
| **GO:0016064 humoral defense mechanism (sensu Vertebrata)** | | | | | |
| 1 | 200986_at | 710 | SERPING1 | serpin peptidase inhibitor, clade G (C1 inhibitor), member 1, (angioedema, hereditary) | 0.00108 |
| 2 | 203104_at | 1436 | CSF1R | colony stimulating factor 1 receptor, formerly McDonough feline sarcoma viral (v-fms) oncogene homol... | 0.0013 |
| 3 | 212067_s_at | 715 | C1R | complement component 1, r subcomponent | 0.0023 |
| 4 | 203854_at | 3426 | CFI | complement factor I | 0.00239 |
| 5 | 209732_at | 9976 | CLEC2B | C-type lectin domain family 2, member B | 0.00633 |
| 6 | 205798_at | 3575 | IL7R | interleukin 7 receptor | 0.00727 |
| 7 | 222292_at | 958 | CD40 | CD40 molecule, TNF receptor superfamily member 5 | 0.00832 |
| 8 | 217767_at | 718 | C3 | complement component 3 | 0.00835 |
| 9 | 206978_at | 1231 | CCR2 | chemokine (C-C motif) receptor 2 | 0.00868 |
| 10 | 208747_s_at | 716 | C1S | complement component 1, s subcomponent | 0.00964 |
| **GO:0006024 glycosaminoglycan biosynthesis** | | | | | |
| 1 | 209834_at | 9469 | CHST3 | carbohydrate (chondroitin 6) sulfotransferase 3 | 0.000114 |
| 2 | 221059_s_at | 4166 | CHST6 | carbohydrate (N-acetylglucosamine 6-O) sulfotransferase 6 | 0.001588 |
| 3 | 218927_s_at | 55501 | CHST12 | carbohydrate (chondroitin 4) sulfotransferase 12 | 0.003358 |
| **GO:0050672 negative regulation of lymphocyte proliferation** | | | | | |
| 1 | 210629_x_at | 7940 | LST1 | leukocyte specific transcript 1 | 0.00195 |
| 2 | 211582_x_at | 7940 | LST1 | leukocyte specific transcript 1 | 0.00817 |
| **GO:0008037 cell recognition** | | | | | |
| 1 | 211571_s_at | 1462 | CSPG2 | chondroitin sulfate proteoglycan 2 (versican) | 0.00163 |
| 2 | 215646_s_at | 1462 | CSPG2 | chondroitin sulfate proteoglycan 2 (versican) | 0.00501 |
| 3 | 204620_s_at | 1462 | CSPG2 | chondroitin sulfate proteoglycan 2 (versican) | 0.00577 |
