# Supplementary figures and images for: Factor interaction analysis for chromosome 8 and DNA methylation alterations highlights innate immune response suppression and cytoskeletal changes in prostate cancer
Source: Mol Cancer. 2007 Feb 5;6:14. doi: 10.1186/1476-4598-6-14 (PMC1797054; doi:10.1186/1476-4598-6-14)

**KLRD1 (p = 0.27)**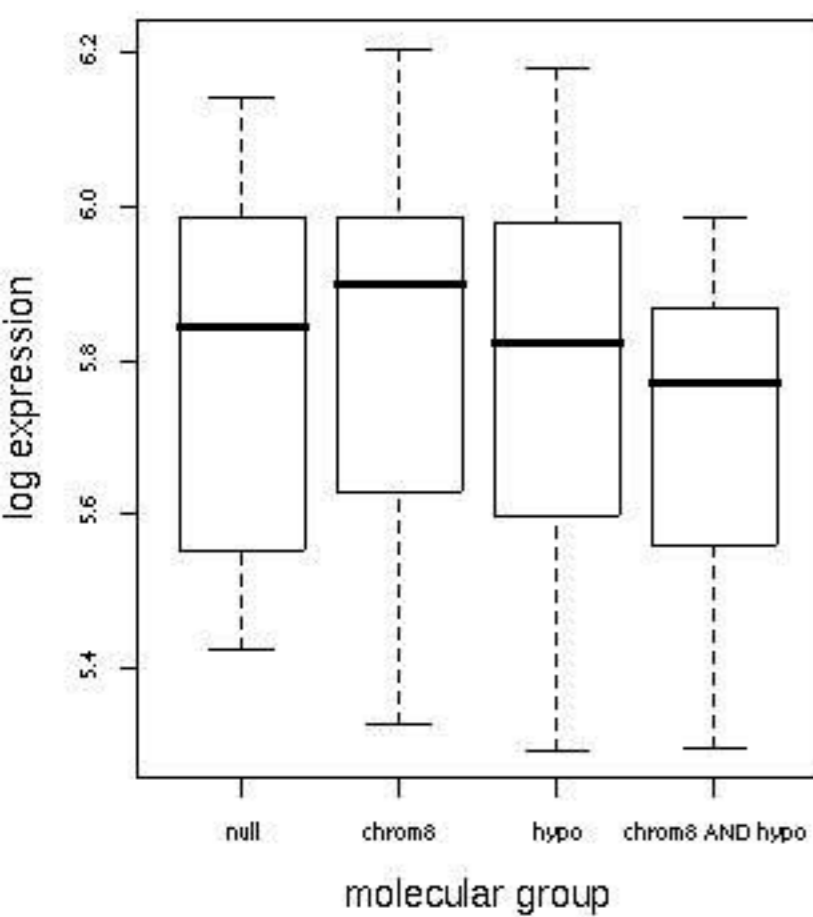**ITGA2 (p = 0.96)**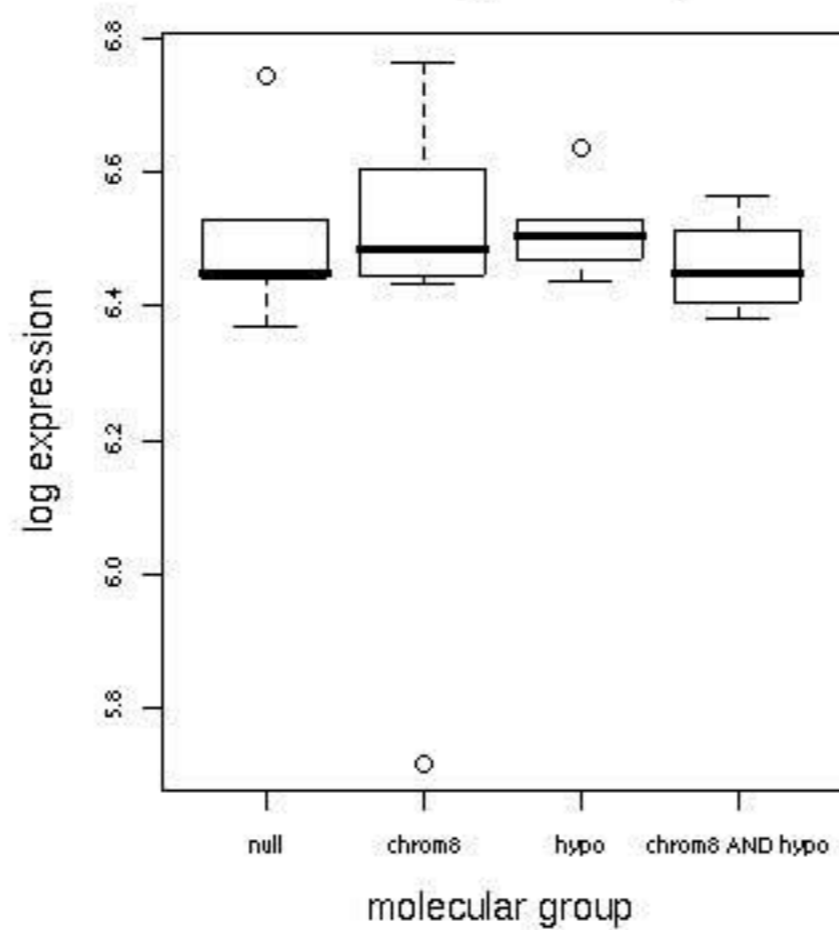**CPA3 (p = 0.12)**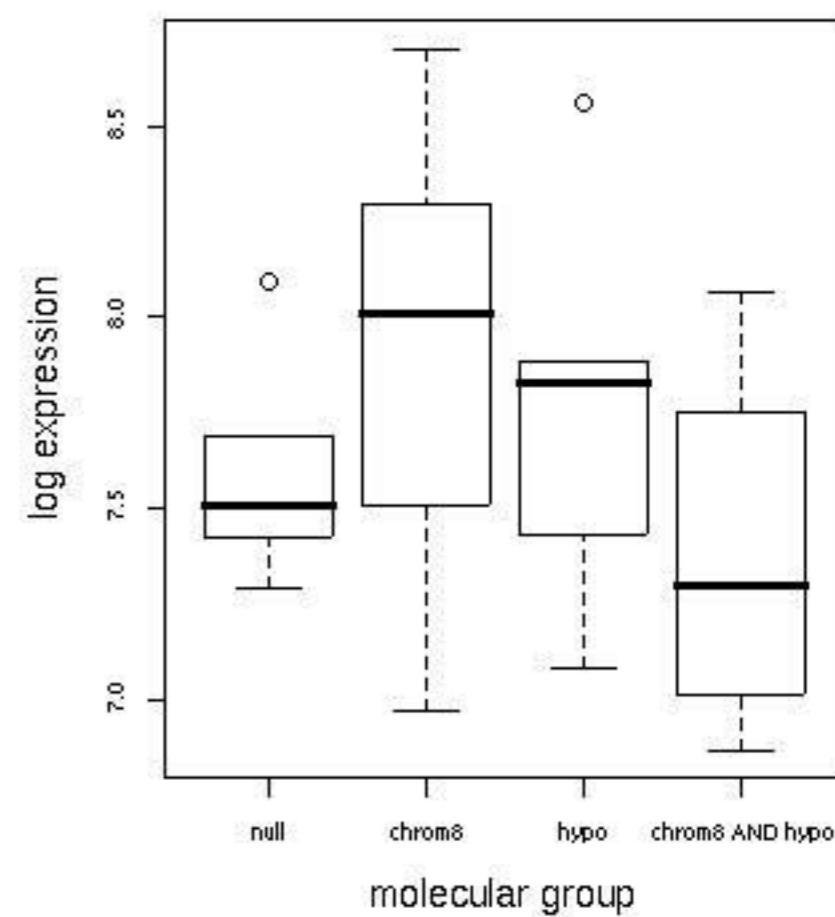**CD163 (p = 0.034)**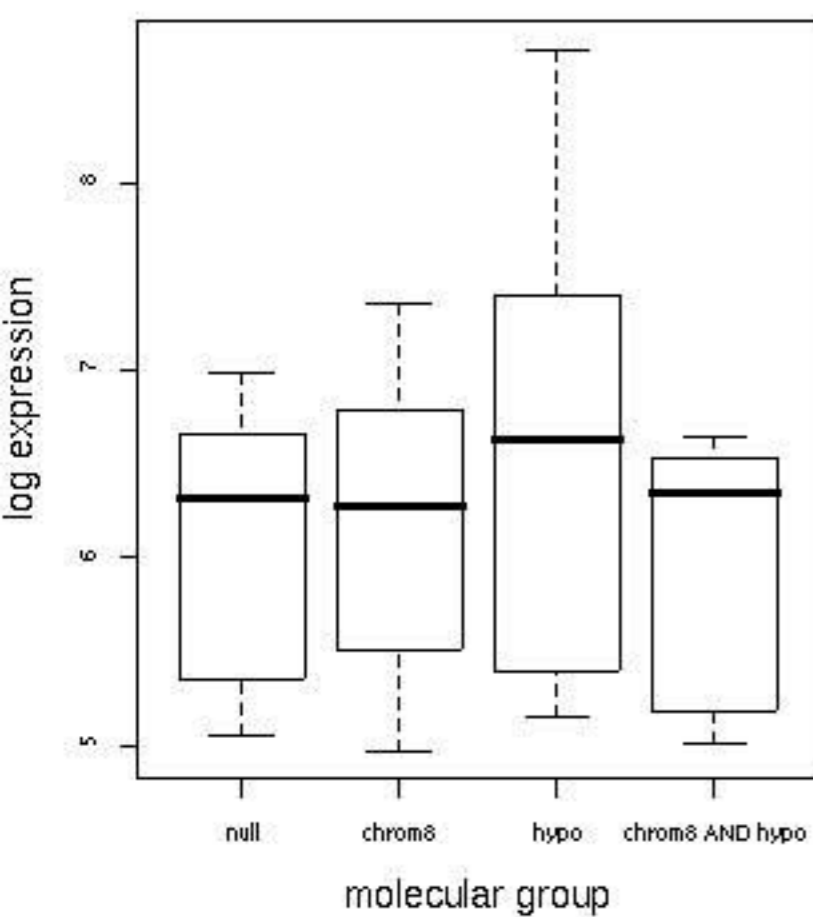**CD83 (p = 0.047)**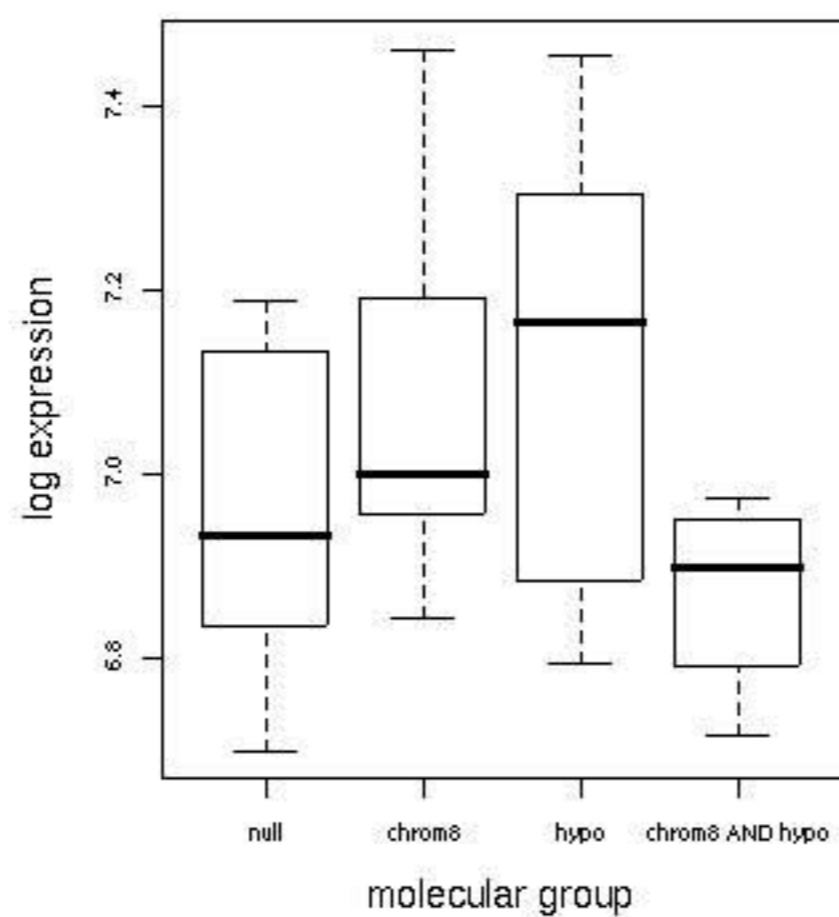**ADAM19 (p = 0.13)**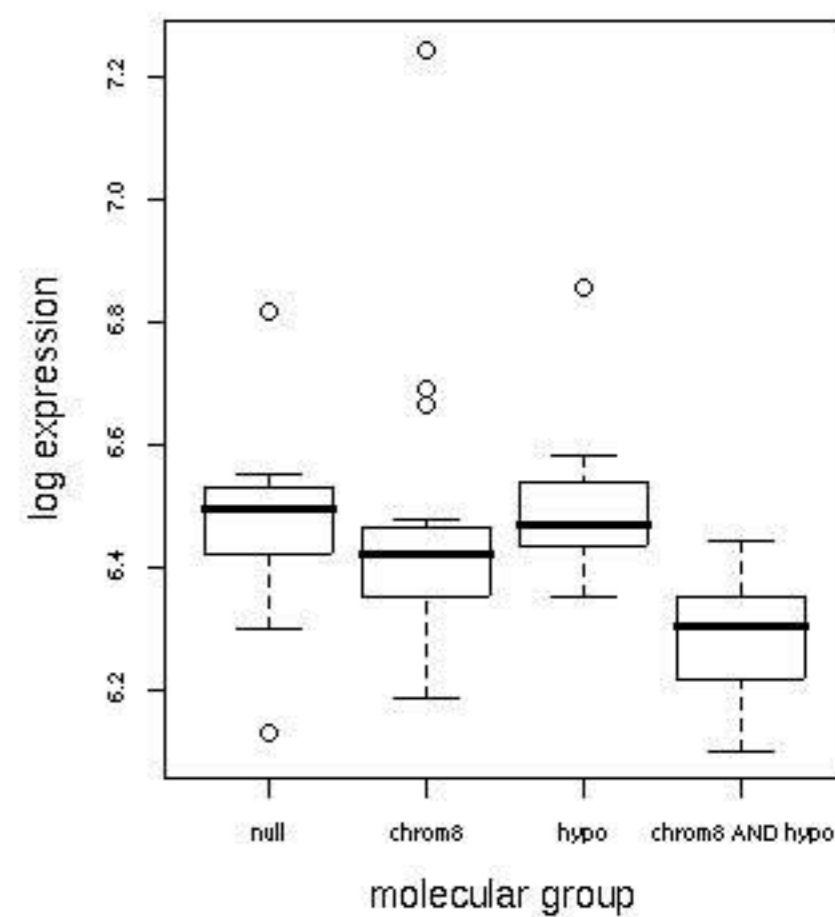**ITGAM (p = 0.0031)**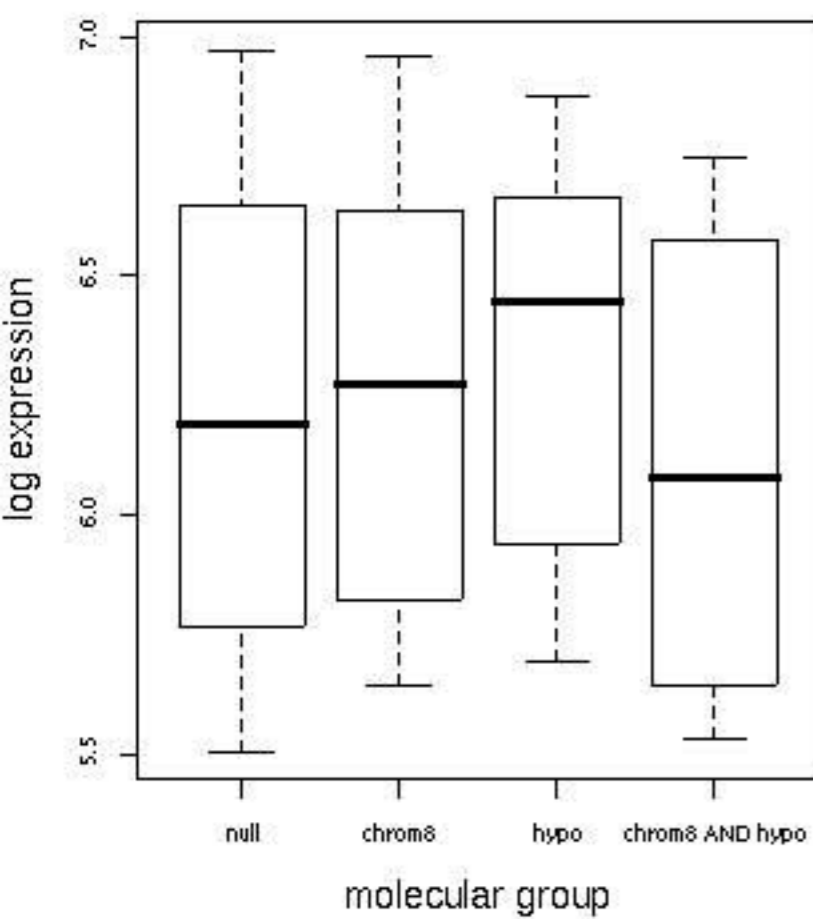

Supplement: Additional file 2 — Box plot representation of microarray analysis results for genes encoding markers of innate immune-response cell types. In each graph, the expression values (log-scale) for the respective genes are depicted for the cancers with neither hypomethylation of LINE-1 retrotransposons nor alteration of chromosome 8 (null), hypomethylation only (hypo), alteration of chromosome 8 only (chrom8) and both alterations (chrom8 AND hypo). The p-values refer to the result of the interaction analysis (see Methods for details). KLRD1 and ITGA2 gene products are characteristic of NK cells, CPA3 of mast cells, CD163 is relatively characteristic of macrophages (the more characteristic marker CD68 was not represented on the HGU133A microarray), CD83 and ADAM19 are typical for dendritic cells, and ITGAM encodes a marker of granulocytes. [file 1476-4598-6-14-S2.pdf]
