# Supplementary material for: Factor interaction analysis for chromosome 8 and DNA methylation alterations highlights innate immune response suppression and cytoskeletal changes in prostate cancer
Source: Mol Cancer. 2007 Feb 5;6:14. doi: 10.1186/1476-4598-6-14 (PMC1797054; doi:10.1186/1476-4598-6-14)
Supplement: Additional file 3 — Additional legend to figure 1. List of GO numbers and GO terms depicted in Fig. 1B and 1C [file 1476-4598-6-14-S3.doc]

**Supplementary legend to Figure 1**. IDs and corresponding term definitions for all GO groups depicted in Figures 1B and 1C

| GO ID | GO term definition |
| --- | --- |
| GO:0001505 | regulation of neurotransmitter levels |
| GO:0001766 | lipid raft polarization |
| GO:0006950 | response to stress |
| GO:0006952 | defense response |
| GO:0006955 | immune response |
| GO:0006956 | complement activation |
| GO:0006959 | humoral immune response |
| GO:0006996 | organelle organization and biogenesis |
| GO:0007009 | plasma membrane organization and biogenesis |
| GO:0007010 | cytoskeleton organization and biogenesis |
| GO:0007154 | cell communication |
| GO:0007155 | cell adhesion |
| GO:0007165 | signal transduction |
| GO:0007267 | cell-cell signaling |
| GO:0007268 | synaptic transmission |
| GO:0007275 | development |
| GO:0007582 | physiological process |
| GO:0008150 | biological process |
| GO:0008152 | metabolism |
| GO:0009056 | catabolism |
| GO:0009605 | response to external stimulus |
| GO:0009607 | response to biotic stimulus |
| GO:0009611 | response to wounding |
| GO:0009613 | response to pest, pathogen or parasite |
| GO:0009790 | embryonic development |
| GO:0009966 | regulation of signal transduction |
| GO:0009967 | positive regulation of signal transduction |
| GO:0009987 | cellular process |
| GO:0016043 | cell organization and biogenesis |
| GO:0016044 | membrane organization and biogenesis |
| GO:0019226 | transmission of nerve impulse |
| GO:0030865 | cortical cytoskeleton organization and biogenesis |
| GO:0031579 | lipid raft organization and biogenesis |
| GO:0031580 | lipid raft distribution |
| GO:0042133 | neurotransmitter metabolism |
| GO:0042135 | neurotransmitter catabolism |
| GO:0044237 | cellular metabolism |
| GO:0044248 | cellular catabolism |
| GO:0045087 | innate immune response |
| GO:0045995 | regulation of embryonic development |
| GO:0048518 | positive regulation of biological process |
| GO:0048522 | positive regulation of cellular process |
| GO:0050789 | regulation of biological process |
| GO:0050793 | regulation of development |
| GO:0050794 | regulation of cellular process |
| GO:0050874 | organismal physiological process |
| GO:0050875 | cellular physiological process |
| GO:0050877 | neurophysiological process |
| GO:0050896 | response to stimulus |
| GO:0051179 | localization |
| GO:0051641 | cellular localization |
| GO:0051665 | lipid raft localization |
| GO:0051668 | localization within membrane |
| GO:0051707 | response to other organism |
